# Microencapsulated α‐Tocopherol and Moringa Extract for Improved Skin Protection: Insights From Human Skin Assessment in Cosmetic Formulations

**DOI:** 10.1111/jocd.70486

**Published:** 2025-10-13

**Authors:** Júlia Cristiê Kessler, Isabel M. Martins, Yaidelin A. Manrique, Sigrún Dögg Gudjónsdóttir, Alírio E. Rodrigues, Maria Filomena Barreiro, Madalena Maria Dias

**Affiliations:** ^1^ LSRE‐LCM, ALiCE, Faculty of Engineering University of Porto Porto Portugal; ^2^ CIMO, LA SusTEC Instituto Politécnico de Bragança Bragança Portugal; ^3^ BIOEFFECT ehf Kópavogur Iceland

**Keywords:** coacervates, permeation, SFE‐CO_2_, skincare

## Abstract

**Background:**

α‐Tocopherol is a potent antioxidant naturally present in human skin, protecting against oxidative damage from UV radiation and pollution. While cosmetic applications may enhance skin protection, its stability and effective delivery remain challenging. *Moringa oleifera* Lam. (Mo) leaves, rich in α‐tocopherol and other bioactives, represent a natural alternative.

**Aims:**

To develop and compare microcapsules containing synthetic α‐tocopherol (MC α‐toc) or a combination of α‐tocopherol with Mo extract (MC Mo + α‐toc), and to evaluate their encapsulation performance, release profile, and efficacy in cosmetic formulations.

**Patients/Methods:**

Mo extract was obtained by supercritical CO₂ extraction, yielding 232.5 ± 3.2 mg α‐tocopherol·g^−1^ extract. Both extracts were microencapsulated by complex coacervation using Arabic gum and gelatine A. Franz cell studies assessed release and absorption compared to free α‐tocopherol. Microcapsules were incorporated into a hydrating cream and tested in a 60‐day study involving 30 healthy Icelandic volunteers. Skin parameters were evaluated against a commercial product.

**Results:**

Microcapsules showed encapsulation efficiency > 93%, loading capacity ~10%, multinucleated morphology, and an average size of 60 μm. Encapsulation enhanced α‐tocopherol absorption, with rates of 6% for MC α‐toc and 3% for MC Mo + α‐toc after 2 h, versus < 1% for nonencapsulated forms. In vivo, MC α‐toc cream reduced skin spots (−25%) and brown spots (−48%), improved firmness (+47%), and increased thickness (+15%). MC Mo + α‐toc cream reduced red areas (−11%) and improved transepidermal water loss (−25%).

**Conclusions:**

Microencapsulation improved stability and delivery of α‐tocopherol and Mo extract, enhancing cosmetic cream performance. Results were comparable or superior to commercial formulations, highlighting their potential for next‐generation skincare products.

## 1 Introduction

Cosmetic formulations are often enriched with functional ingredients designed to enhance protection and restore the integrity of the epidermal and dermal barriers [1]. Common functional ingredients include antioxidant and anti‐inflammatory agents, which help to combat oxidative stress, improve hydration, and reduce irritation [2, 3]. These ingredients synergistically support skin health, promote tissue repair, and maintain a balanced skin barrier function.

**FIGURE 1:**
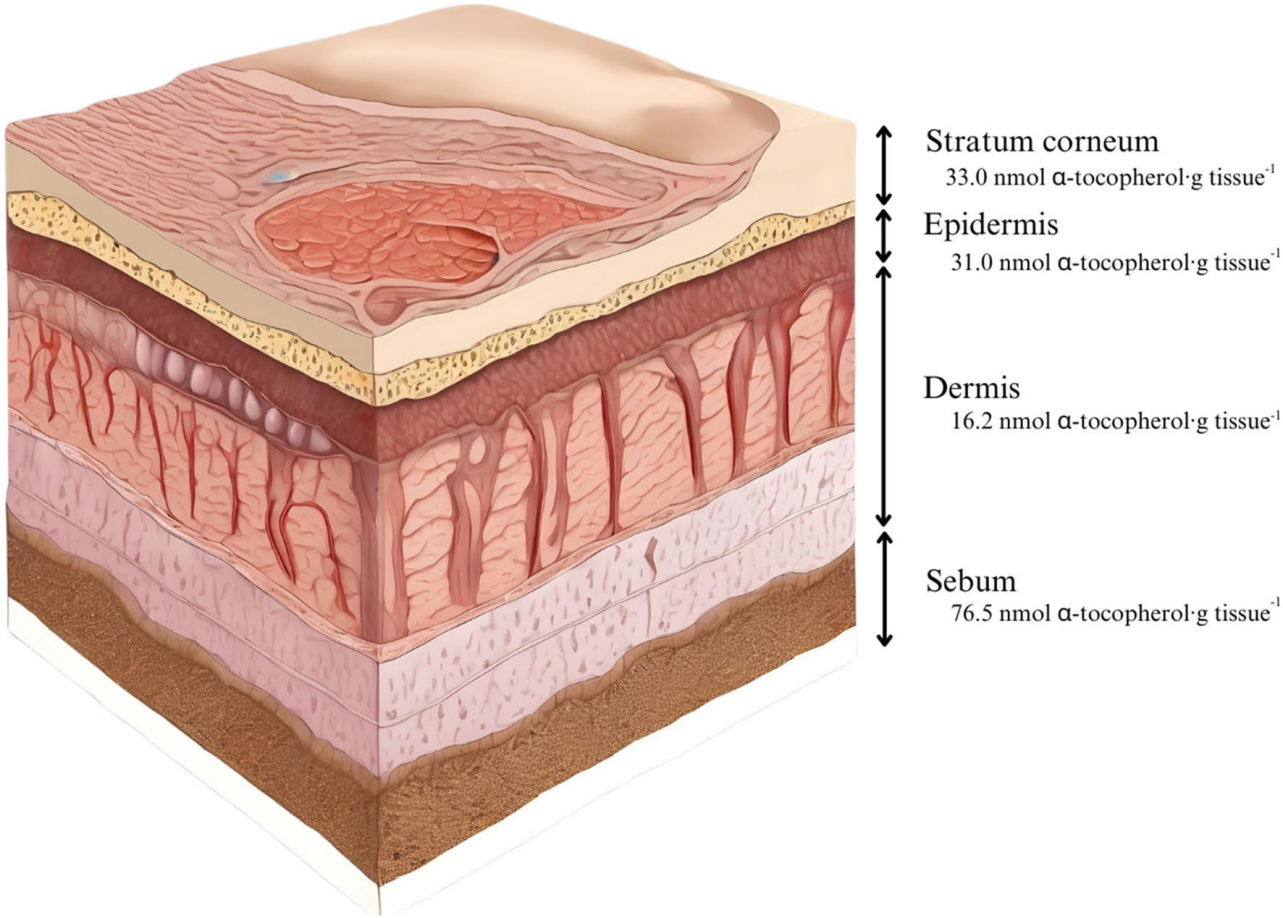
Illustration of human skin structure and respective α‐tocopherol distribution. Image generated by AI [7]. Data extracted from [4].

α‐Tocopherol is the main component of vitamin E and the most abundant lipophilic antioxidant found in human skin, significantly mitigating acute skin reactions caused by repeated exposure to prooxidative environments, such as ultraviolet (UV) radiation, drugs, and air pollution [4]. Endogenous reactive oxygen species (ROS) and other free radicals also contribute to skin oxidative damage, which becomes more pronounced with aging, disrupting the balance between α‐tocopherol and skin oxidative stress [5, 6]. Figure 1 shows the layered skin structure, with α‐tocopherol concentrations of circa 33.0 nmol_α‐tocopherol_·g_tissue_^−1^ in the stratum corneum, 31.0 nmol·g^−1^ in the epidermis, 16.2 nmol·g^−1^ in the dermis, and 76.5 nmol·g^−1^ in the sebum. The continuous production of α‐tocopherol is stimulated by oral intake of foods rich in this nutrient (e.g., nuts and seeds) and can be further enhanced by topical applications [4].

Concentrations of α‐tocopherol ranging from 0.1% to 1% are considered safe and effective in enhancing antioxidant protection [4]. However, according to the Personal Care Products Council (Council), the concentration of α‐tocopherol in leave‐on products has increased significantly [8]. The last released data shows a rise from 2% in 1999 to 5.4% in 2013. This increase reflects the absence of strict efficacy limits for non‐pharmaceutical ingredients such as α‐tocopherol, despite its designation as Generally Recognized as Safe (GRAS) by the Food and Drug Administration (FDA) [4, 8]. This trend underscores the importance of regulatory frameworks such as Regulation (EC) No 1223/2009 [9], which governs cosmetic products in the European Union to ensure their safety, efficacy, and proper labeling.

Using synthetic and natural extracts in cosmetic formulations provides a balanced approach to enhancing skincare efficacy. Synthetic compounds are engineered for stability and targeted effects, such as anti‐aging and skin renewal; they may occasionally cause adverse reactions, including allergic and irritant contact dermatitis and phototoxic or photo‐allergic responses [10]. In contrast, natural extracts offer a complex blend of bioactive components—antioxidants, vitamins, and minerals—that promote skin health and disease prevention while being hypoallergenic and easily absorbed by the epidermis [10, 11]. By combining synthetic and natural ingredients, formulations can achieve synergistic effects, where the stability and precision of synthetic compounds complement the bioavailability and safety of natural extracts.

The potential of *Moringa oleifera* Lam. (Mo) extracts for skincare applications, with a particular focus on the advantages of extraction by supercritical fluid extraction with carbon dioxide (SFE‐CO_2_), recognized by its high compound selectivity, was the subject of a recent review [12]. In another study, Mo leaf extract (Mo) was found to contain up to 21% in relative composition of α‐tocopherol, equivalent to 148 mg_compound_·g_extract_^−1^, and other hydrophobic functional compounds in samples harvested in 2021 [12]. Higher selectivity was obtained using SFE‐CO_2_ under optimized conditions of 195 bar and 55°C for 120 min, yielding promising ingredients suitable for cosmetic formulations.

Bioactives can be freely dispersed into the formulation or incorporated into a delivery system, such as microcapsules, to protect the active compound and facilitate controlled release. These systems enhance the stability of the bioactive and ensure more effective delivery within the formulation [13]. Microencapsulation by complex coacervation (MCC) is a well‐established technique used to encapsulate a wide range of compounds, providing stability and controlled release with high encapsulation efficiency. The coacervation process usually occurs between two polymeric solutions with opposite superficial charges, and it is strongly influenced by factors such as pH, ionic strength, and polyion concentration [14]. Recently, an MCC system tailored for cosmetic ingredients was developed using Arabic gum (AG) and gelatine A (GE) as the coacervating agents, producing microcapsules approximately 60 μm in size, with up to 90% efficiency and multinucleated morphology [15]. These microcapsules were designed to entrap various lipophilic molecules, including α‐tocopherol and other compounds identified in Mo extracts.

Despite their potential to penetrate the dermal layers and their widespread use in topical formulations, the mechanisms by which lipophilic active compounds penetrate human skin remain incompletely understood [4]. The transdermal permeation of α‐tocopherol is particularly challenging due to its high hydrophobicity and large molecular mass [16]. Previous studies with oil‐in‐water (o/w) cosmetic formulations containing α‐tocopherol applied to reconstructed human epidermis (RHE) and synthetic membranes in Franz diffusion cells demonstrated a prolonged release lag time, with α‐tocopherol appearing in the receptor fluid only after 22 h [17].

To better understand α‐tocopherol's penetration, this study presents a comprehensive development of cosmetic formulations enriched with microcapsules, offering new insights into permeation mechanisms and human skin assessment. It takes a pioneering approach, first addressing the bioactive extraction and quantification of Mo harvested in 2023 using optimized SFE‐CO_2_ conditions [12] for advanced skincare applications. By integrating optimized bioactive extraction and MCC, this study validates a previously developed delivery system [15] encapsulating α‐tocopherol (MC α‐toc) and a combination of Mo extracts and α‐tocopherol (MC Mo + α‐toc) as active materials.

The study further evaluates these microcapsules through release tests using synthetic membranes that mimic human skin, comparing their performance to non‐microencapsulated counterparts (in solution). Finally, both microcapsules were incorporated into an adapted formulation of a hydrating cream from the cosmetics company BIOEFFECT ehf. (bioeffect.com, Reykjavík, Iceland), and their efficacy was assessed in a 60‐day human trial involving 30 volunteers, benchmarking them against a leading commercial product.

This study provides a scientifically validated pathway for enhancing α‐tocopherol skin penetration. The findings not only demonstrate the efficacy of these microcapsules in real‐world cosmetic formulations but also highlight their potential as a sustainable and innovative alternative to conventional skincare ingredients—paving the way for the next generation of high‐performance, naturally derived cosmetics.

## 2 *Moringa oleifera* Lam. Leaves Extract

### 2.1 Materials

Mo leaves were sourced from Moringa del Sur (moringadelsur.com, Malaga, Spain) and harvested in May 2023. The sample preparation consisted of drying the leaves at room temperature and grinding them into particle sizes ranging between 0.50 and 0.70 mm, using an Hr7762/90 Mini Chopper (Philips Walita, Amsterdam, The Netherlands) for 20 s.

Carbon dioxide (CAS 124‐38‐9, food grade, < 99%) was purchased from Air Liquid (Paris, France), ethanol absolute (CAS 64‐78‐6, ≥ 99%) from Supelco (Madrid, Spain), and n‐hexane (purity 95% HPLC, CAS 110‐54‐3) from VWR (Lisbon, Portugal), respectively.

### 2.2 SFE‐CO_2_ Extraction

Mo leaf extracts were obtained using a previously described bench‐scale SFE‐CO_2_ equipment under optimized conditions, determined through a screening study that identified the optimal combination of pressure and temperature for enhanced α‐tocopherol extraction selectivity [12]. Briefly, the extraction was carried out with a CO_2_ flow rate of 4 mL·min^−1^, at 195 bar and 55°C for 120 min, using 10 g of raw material per extraction. The extract was collected into a separator at pressure and temperature below 40 bar and 5°C. Finally, the apparatus was cleaned using 200 mL of ethanol, and CO_2_ was pumped for 15 min to ensure total yield recovery.

For extract analysis and subsequent extract microencapsulation, deionized water was added to the ethanolic solution in a 50:50 ratio and subjected to freeze‐drying (55C, CoolSafe, Beverwijk, The Netherlands) at −55°C until constant weight to remove all liquid. The solid extract was stored in occlusive conditions at 7°C for further use.

### 2.3 Extract Characterization

The chemical composition of Mo leaves extract was evaluated using gas chromatography (TQ8040 NX Triple Quadrupole, Shimadzu, Kyoto, Japan) coupled with an ion‐trap mass spectrometer (GC–MS). The equipment operated in splitless injector mode, with a cross‐bonded fused column (30 m × 0.25 mm, 0.25 μm film thickness) for low‐polarity phases (Rxi‐5Sil MS, Restek, Bellefonte, PA, USA) and an automatic sampler (AOC‐20s + i).

Each sample was injected at 280°C with a 1 μL injection volume, using ultrapure helium as the carrier gas at a flow rate of 1 mL·min^−1^ under linear velocity flow control. The mass spectrometer operated with a scanning range of m/z 40–500, while the ion source and interface temperatures were maintained at 250°C and 260°C, respectively.

The oven temperature program for Mo leaf extracts was designed to ensure optimal compound separation. Initially, the oven was maintained at 40°C for 1 min, then heated to 200°C for 2 min at a rate of 7°C·min^−1^. This was followed by a temperature increase to 250°C for 2 min at 15°C·min^−1^, before finally stabilizing at 280°C for 1 min at 20°C·min^−1^.

Mass spectra were compared against reference data in the National Institute of Standards & Technology (NIST 21, 27, 107, 147) database. Linear retention indices (LRI) were determined using Kovats retention index calculations, based on a homologous series of alkanes (C8–C20) analyzed under identical chromatographic conditions [12]. α‐Tocopherol was quantified through a calibration curve ($y=1.04\times 10^{10}x–7.87\times 10^{6}$, *R* ^2^ = 0.9996).

### 2.4 The Extract

Table 1 shows the chemical composition of the obtained Mo leaf extract. A total of 21 compounds were identified. The target bioactive compound, α‐tocopherol, was found to comprise 8.43% ± 0.09% of the relative composition, representing 232.5 ± 3.2 mg_α‐tocopherol_⋅g_extract_^−1^.

**TABLE 1:** Chemical profile of *Moringa oleifera* Lam. leaf extract obtained by SFE‐CO_2_.

Chemical group	Compound (order of detection)	Base peak	RT (min)	LRI	Relative concentration (%)	± SD (%)
Adehydes	Pentadecanal (**13**)	82, 57, 43	33.436	2838	1.27	0.05
Fatty acids	Palmitic acid (**3**)	88, 101, 43	24.560	1992	8.25	0.13
	Oleic acid (**5**)	55, 41, 69	26.930	2137	1.80	0.24
	Linolenic acid, methyl ester (**6**)	67, 81, 95	27.246	2161	5.05	0.45
	Linolenic acid, ethyl ester (**7**)	79, 95, 67	27.324	2167	0.47	0.08
	Ethyl pentadecanoate (**8**)	88, 101, 43	27.669	2194	0.64	0.06
	Arachidic acid (**11**)	88, 43 101	33.108	2795	0.58	0.01
Alcohols	1‐Tetracosanol (**21**)	57, 97, 83	38.071	3300	26.35	0.88
Hydrocarbons	8‐Hexylpentadecane (**9**)	57, 71, 43	30.525	2500	2.59	0.03
	Heptacosane (**10**)	57, 71, 43	32.436	2701	10.33	0.17
	Squalane (**12**)	57, 71, 85	33.141	2800	0.94	0.05
	Nonacosane (**14**)	57, 71, 43	33.926	2901	19.67	0.08
Terpenes	*cis*‐Phytol (**1**)	81, 82, 43	22.290	1835	0.19	0.01
	*trans*‐Phytol (**4**)	71, 57, 43	26.522	2106	3.04	0.03
Tocopherols	γ‐Tocopherol (**16**)	151, 416, 191	35.289	3051	0.56	0.01
	Vitamin E (α‐Tocopherol) (**19**)	165, 430, 164	36.081	3129	8.43	0.09
Vitamins	l‐(+)‐Ascorbic acid 2,6‐dihexadecanoate (**2**)	57, 43, 73	24.195	1967	1.18	0.04
Unknowns	Unknown (**15**)		35.202	3042	3.38	0.02
	Unknown (**17**)		35.658	3089	2.52	0.02
	Unknown (**18**)		35.728	3096	1.84	0.01
	Unknown (**20**)		36.125	3133	1.01	0.01
Identified					**90.92**	
**α‐tocopherol**	**Calibration curve**	***R*** ^**2**^	**LOD (g⋅L** ^**−1**^)	**LOQ (g⋅L** ^**−1**^)	**mg**_**α‐tocopherol**_ **⋅ g**_**extract**_^**−1**^	
	$y=1.04\times 10^{10}x–7.87\times 10^{6}$	0.9996	$6.18\times 10^{-4}$	$1.87\times 10^{-3}$	232.5 ± 3.2	

*Note:* LRI: linear retention indices calculated through Kovats retention index equation for series of alkanes C8‐C10 using a cross‐bonded fused column in GC–MS. The bold values in Table 1 correspond to the statistical parameters R² (coefficient of determination), LOD (limit of detection), and LOQ (limit of quantification).

Abbreviations: R² – Coefficient of determination; LOD – Limit of detection; LOQ – Limit of quantification.

Different values were reported in the previous optimization study, specifically 21.5% ± 0.8% and 148.0 ± 4.9 mg⋅g^−1^ [12]. In both cases, the crop material was harvested from the same location during May 2021 and 2023, respectively, and submitted to a similar drying process at room temperature. Therefore, the differences in composition can be mainly attributed to the influence of climate, which affects the plant's metabolism and bioactives' availability for extraction [18]. For example, temperature and light intensity play an important role in the antioxidant response to environmental adaptations. Exposure to high light and heat stress, or to high light and low temperature, can lead to the accumulation of high levels of α‐tocopherol or can cause lipid peroxidation [19]. Additionally, drought periods have also been mentioned as significant stressors affecting both yield and extract composition [20].

Overall, the extracts have proven to be a reliable source of α‐tocopherol, alongside other compounds of interest for cosmetic products, including nonacosane (19.7% ± 0.1%). Nonacosane, a hydrocarbon with emollient properties found on the leaf surface, is easily extracted due to its low mass transfer resistance to supercritical CO_2_ [21]. This compound presents a predominantly solid waxy appearance with hydrophobic characteristics, as observed in the Mo extracts.

## 3 Extract Stabilization Using Microencapsulation by Complex Coacervation

### 3.1 Reactants

Gelatine A (GE, from porcine skin, 300 g bloom, CAS 9000‐70‐8), Arabic gum (AG, from Acacia tree, CAS 90000‐01‐5), N‐hydroxy succinimide (NHS, purity ≥ 97%, CAS 6066‐82‐6), Polysorbate 80 (P80, HLB 4.3, CAS 9005‐65‐6), and hydrogen chloride (HCl, purity ≥ 37%, CAS 7647‐01‐0) were purchased from Sigma Aldrich (Madrid, Spain). 1‐ethyl‐3‐(3‐dimethylaminopropyl) carbodiimide (EDC, purity ≥ 98%, CAS 25952‐53‐8) was acquired from Alfa Aesar (Budapest, Hungary). Caprylic triglyceride (CAP, from Cocos mucifera oil, batch 14 832) was purchased from Plena Natura (Lisbon, Portugal), while α‐tocopherol (CAS 10191‐41‐0, 99%) was acquired from BASF (Ludwigshafen, Germany).

### 3.2 Microcapsules Production

The methodology to produce the microcapsules designed for cosmetic applications was previously optimized using a two‐fold fractional factorial Design of Experiments, varying the carrier‐to‐emulsifier ratio (CAP:P80), stirring rate and time, and crosslinker concentration (EDC/NHS). No active ingredient was used in the optimization study [15]. These parameters were applied here for the encapsulation of α‐tocopherol and Mo extract. Briefly, MCC in batch mode was used to enclose the bioactives. The process begins with emulsification, where 9 g of the oil phase—comprising to the core (α‐tocopherol, Mo extract, and CAP) and the emulsifier (P80) in a 3.5:1 ratio—is mixed with 50 mL of GE aqueous solution (7.14 g L^−1^) at 9500 rpm for 2 min using an Ultra Turrax (IKA Yellowline DI 25, Gravimetra, Lisbon). The next steps occur under continuous stirring at 400 rpm with a magnetic stirrer (Agimatic‐N, J.P. Selecta, Barcelona). Polymer addition follows, where the emulsion is transferred to a beaker, and 50 mL of GA aqueous solution at 14.30 g L^−1^ is gradually added dropwise over 4 min, then stirred for an additional 6 min at 40°C to stabilize the oil droplet cores. During coacervation, the pH of the dispersion is lowered from 5.4 to 3.9 by adding 1 M HCl using a pH meter (Hanna, HI 8424, Smithfield, United States). The mixture is then cooled in an ice bath to 7°C for 30 min. Hardening step is carried out by adding 10% of the total volume in EDC 50 mM and NHS 25 mM in equal amounts to crosslink the coacervates under stirring for 60 min. The crosslinked dispersion undergoes overnight decantation under refrigeration at 7°C. Finally, the microcapsules are collected and washed twice with 60 mL of deionized water at 40°C to remove any non‐reacted reagents.

Table 2 summarizes the core material ratio used for each formulation, with the amount of CAP as a reference.

**TABLE 2:** Characteristics of the microcapsules produced by coacervation containing α‐tocopherol and *Moringa oleifera* Lam. leaf extract.

Test	α‐tocopherol (%)	Mo extract (%)	Mean particle size (μm)	SC (%)	EE (%)	LC (%)	pH
MC α‐toc	20.0	—	60.0	8.3 ± 0.1a	95.1 ± 1.2a	10.3 ± 1.2a	4.4a
MC Mo + α‐toc	19.6	0.4	61.2	8.4 ± 0.5a	93.4 ± 1.3a	10.0 ± 1.3a	4.5a

*Note:* Different letters in the same column represent significant differences (α = 0.05).

### 3.3 Microcapsules Characterization

#### 3.3.1 Particle Shape and Configuration

The shape and configuration of MC α‐toc and MC Mo + α‐toc dispersions were assessed using an Optical Microscopy (Eclipse Ci H55OS, equipped with DS‐Qi2 camera, Nikon, Tokyo, Japan) and the images were treated using NIS‐Elements L software. The analyses were performed at 10× and 40× magnifications.

#### 3.3.2 Encapsulation Efficiency and Loading Content

The sample preparation for measuring the encapsulation efficiency (EE%) of α‐tocopherol consisted in taking 2 mL aliquot of the homogenized formulation, combining with 1 mL of hexane and centrifuged (Micro Star 30, VWR, Pennsylvania, USA) at 3000 rpm (80.5 g) for 5 min. The supernatant was collected, filtered through a 0.22 μm PTFE hydrophobic syringe filter (25 mm, CAT 28145‐495, VWR, Pennsylvania, USA), and transferred to the injection vial [15]. For loading content (LC%) analysis, 5 mL of microcapsule dispersion was weighed before and after freeze‐drying at −55°C for 48 h (CoolSafe, Beverwijk, The Netherlands). The resulting solid microcapsules were disrupted with 1 mL of HCl and sonicated for 30 min [22]. Then, liquid–liquid extraction was performed using 2 mL of hexane, followed by centrifugation, filtration, and transference to GC–MS vials for analysis.

EE% and LC% quantification settings operated at the same conditions using an isothermal programme at 40°C for 1 min, then raised from 40°C to 200°C for 1 min at 30°C·min^−1^, 200°C to 250°C for 2 min at 5°C·min^−1^, followed by an increase to 280°C for 2 min at 1°C·min^−1^ and, finally, 300°C for 2 min at 3°C·min^−1^ for total compound separation. Ultrapure helium, flowing at 1 mL·min^−1^, was injected with 1 μL of the sample at 280°C under linear velocity control mode with a split ratio 2. The analysis was conducted with mass scanning set to m/z 20–500, and the ion source and interface temperatures were maintained at 250°C and 260°C, respectively.

Each molecule was identified by comparing the mass spectra of the molecules to those in the National Institute of Standards and Technology (NIST) database (21, 27, 107, and 147). Their linear retention indices (LRI) were calculated using the Kovats retention index equation, utilizing a homologous series of alkanes (C8–C20) analyzed under the same chromatographic conditions. α‐tocopherol was quantified using a calibration curve based on its respective analytical standard ($y=1.04\times 10^{10}x–7.87\times 10^{6}$, *R* ^2^ = 0.9996).

The samples were analyzed in triplicate, and the data was used for the indirect determination of the encapsulation efficiency,

(1) $$\mathrm{EE}\;\left(\%\right)=\frac{\text{total mass}-\text{nonencapsulated mass}}{\text{total mass}}\times 100$$

while the loading content was calculated by

(2) $$\mathrm{LC}\;\left(\%\right)=\frac{\text{total}\;\alpha -\text{tocopherol mass}}{\text{total microcapsules mass}}\times 100$$

#### 3.3.3 Solid Content

The microcapsule dispersions were submitted to an air‐drying process at 105°C ± 1°C until a constant weight was achieved. This process was used to measure the solid content (SC%) gravimetrically, determined by [23]:

(3) $$\mathrm{SC}\;\left(\%\right)=\frac{\mathrm{dry}\;\text{mass}}{\text{liquid mass}}\times 100$$

#### 3.3.4 Statistical Assessment

The significant differences were assessed by ANOVA (Analysis of Variance) and Tukey tests using Statistica StatSoft (version 14, United States), with α = 0.05 (significance level; 95% confidence).

### 3.4 The Microcapsules

Microcapsules optimized and tailored for cosmetic formulations were used in this study to incorporate both α‐tocopherol and Mo extracts, resulting in the production of two types of microcapsules, designated as MC α‐toc and MC Mo + α‐toc, respectively.

Table 2 summarizes the conditions and main characteristics obtained for the coacervates. The core material was composed of carrier (CAP) and respective bioactives. About 20% of the oily phase was bioactive, with synthetic tocopherol in both formulations. A fraction of 0.4% of Mo extract was used in MC Mo + α‐toc, highly limited by its solubility and volume into CAP. Mo extract was added freeze‐dried, exhibiting notable hydrophobicity, as mentioned in section 2.4.

**FIGURE 2:**
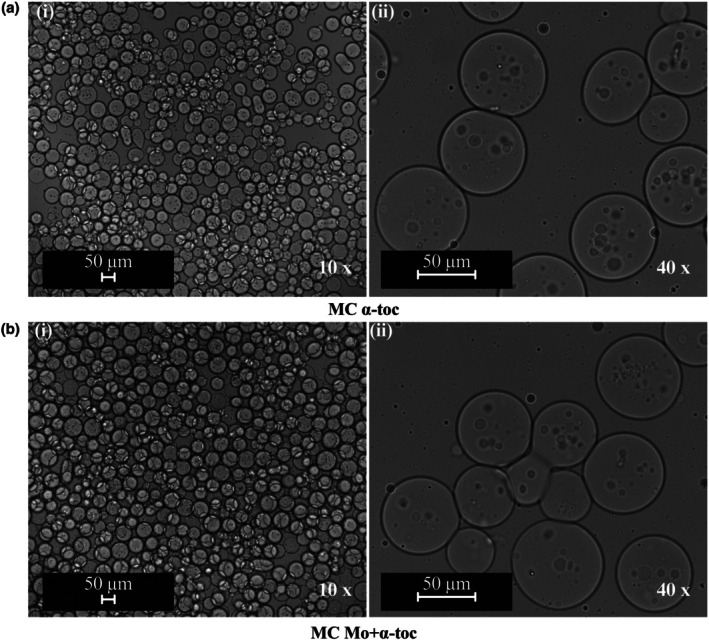
Optical microscopy of microcapsules containing (a) α‐tocopherol (MC α‐toc) and (b) *Moringa oleifera* Lam. extract (MC Mo + α‐toc).

Figure 2 shows optical images of the developed microcapsules, revealing a spherical shape with multinucleated morphology in both cases. The coacervate system optimized by [15] exhibited similar characteristics, with a polymeric outer layer showing a honeycomb‐like structure, confirmed through Confocal and CryoSEM imaging analyses. The microcapsules have an average size of approximately 60 μm, a moisture content of circa 8.3%, and pH in the range 4.4–4.5 (see Table 2). There were no significant differences between the tested core materials (α = 0.05), consistent with the previously designed microcapsules [15].

Encapsulation efficiency between 93% and 95% and loading content around 10.3% (Table 2) reflect the known high performance of this MCC system in coating hydrophobic bioactives [24], surpassing those reported for other AG/GE microcapsules [25, 26]. Although the LC% appears low compared to, for example, black raspberry microcapsules (38.5%) [27], this study used approximately 33 times less active material for coacervation, a considerable difference in shell‐to‐core material ratio. Additionally, further analysis of the ratio indicated that the LC could not surpass 14%. To date, no other study has reported the microencapsulation of α‐tocopherol or extract‐rich formulations using AG/GE coacervates.

## 4 Release Tests

### 4.1 Materials

Strat‐M, 25 mm, PES membrane for transdermal release testing, was acquired from MilliporeSigma (Darmstadt, Germany); disodium phosphate (Na_2_HPO_4_, ≥ 99%, CAS 7558‐79‐4), and monopotassium phosphate (KHPO_4_, ≥ 99%, CAS 7778‐77‐0) were obtained from VWR (Suwon, Japan); potassium chloride (KCl, CAS 7447‐40‐7) was purchased from AppliChem GmbH (Darmstadt, Germany); polyoxyethylene sorbitan trioleate (CAS 9005‐70‐3) and isopropanol (99.9%, CAS 67‐63‐0) were acquired from Merck (Madrid, Spain) and Sigma Aldrich (Darmstadt, Germany), respectively.

### 4.2 Franz Cell Diffusion Tests

The release tests were conducted using static vertical glass Franz diffusion cells with a donor area of 0.64 cm^2^ and a receptor chamber of 5.0 mL (Permegear Inc., Hellertown, United States). Strat‐M membranes were utilized as a substitute for human skin, in alignment with the European Commission's regulations prohibiting animal testing in cosmetics [28]. These synthetic membranes closely replicate human skin and have demonstrated consistent reliability and effectiveness in permeability assessments [29, 30, 31].

The membrane was placed facing the receptor fluid, a PBS buffer solution (pH 7.4), maintained at 37°C [32, 33] using a heating bath circulator thermostat (Huber Pilot ONE, Hanover, Pennsylvania, United States), and continuously stirred with a magnetic stirrer at 600 rpm under occlusive conditions.

In this setup, the skin surface is represented by the donor chamber, the stratum corneum and epidermis by the membrane, and the dermis by the receptor chamber.

To evaluate the systems' performance over 1, 2, 4, 8, and 24 h of experimentation, modifications were made to the standard method for individual batches of analyzes [34]. Originally, the method consists of taking aliquots from the receptor fluid for analysis over time. However, this approach failed to detect α‐tocopherol due to its very low concentration. In contrast, analyzing the individual components at each time point and reconstructing the system under the sample conditions provided a complete understanding of the α‐tocopherol's release.

Therefore, the release tests were performed for both MC α‐toc and MC Mo + α‐toc microcapsules, as well as for the bioactives in their free form solubilized in CAP. Each time, the donor chamber was filled with 300 μL of sample. The structure was carefully disassembled at the end of the analysis period, and samples from the different parts of the Franz cell were collected.

The sample remaining on the upper layer of the membrane was washed off and collected using 6 mL of hexane. The bioactive solutions were then filtered and transferred to GC–MS vials for analysis. In the case of the microcapsule dispersion, both the bioactives remaining in the donor chamber but not absorbed by the membrane and those still encapsulated were analyzed. For this, a 1 mL aliquot of the microcapsule solution followed the same procedure as the bioactive solutions, while the last 5 mL was disrupted by mixing with 1 mL of HCl, sonicated for 30 min, filtered, and diluted 10‐fold for analysis.

The α‐tocopherol absorbed by the membrane was collected using a solid–liquid extraction with 3 mL of hexane, followed by 30 min of sonication, filtration, and dilution for GC–MS analysis.

Finally, to assess the permeated content in the receptor fluid, the entire volume from the receptor chamber was collected, and 2 mL of hexane was added for liquid–liquid extraction. The mixture was centrifuged at 3000 rpm for 5 min, and the resulting supernatant was filtered and analyzed.

The mass quantification was carried out according to the protocol described in section 3.3, and the data were used to validate the presence of the compounds through a mass balance, given by

(4) A=A*+B+C

where A is the total initial mass, A* is the retained mass into the donor chamber over time, B is the absorbed mass by the membrane over time, and C is the mass permeated into the receptor fluid over time.

According to the Scientific Committee on Consumer Safety (SCCS) guidelines, the mass balance may achieve an overall range between 85% and 115% [35].

The statistical significances of the extracts and microcapsules were evaluated as described in section 3.3.

### 4.3 The Release Behavior

**FIGURE 3:**
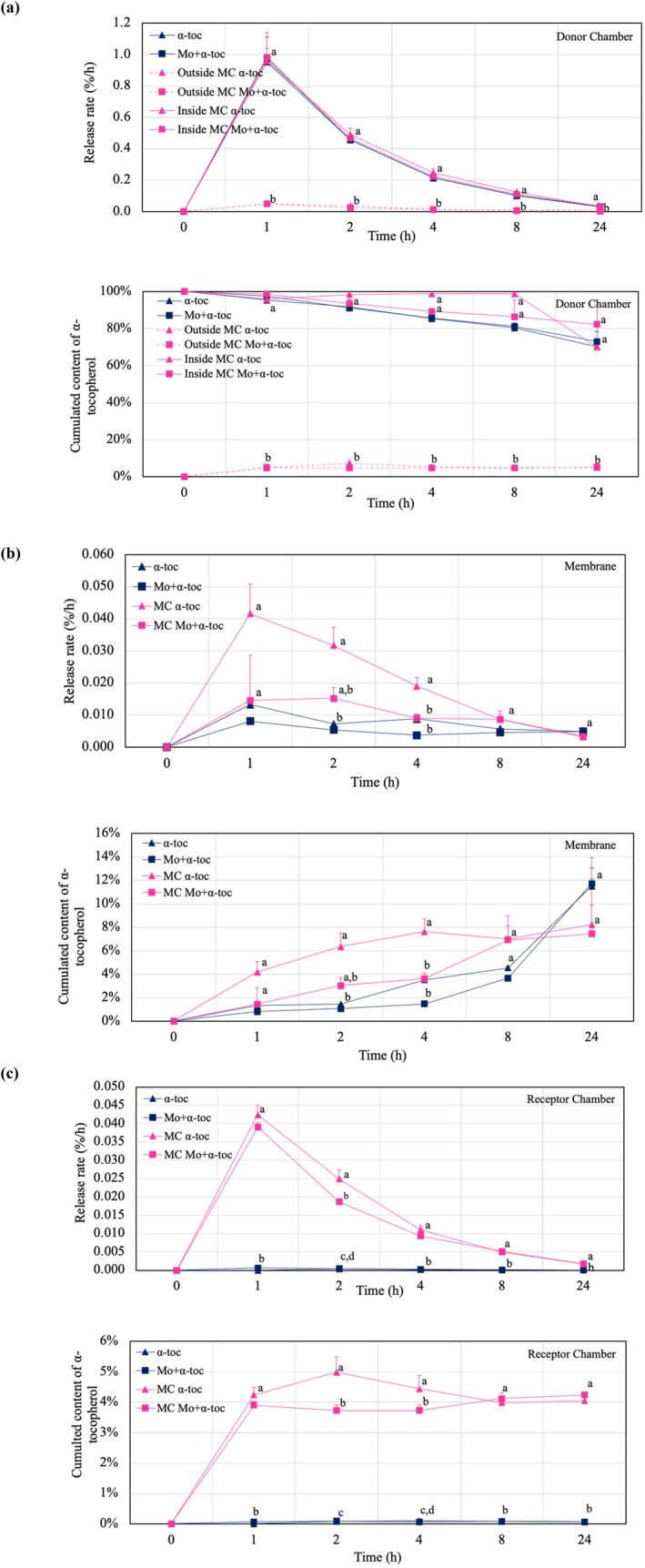
Release rate and cumulated content of EGF in solution quantified in the (a) donor chamber, (b) membrane, and (c) receptor chamber over time. Different letters in the same curve represent significant differences (α = 0.05).

Results of Franz cell release tests for MC α‐toc and MC Mo + α‐toc and the free bioactives in solution are shown in Figure 3. In the following discussion, the terminology used to describe each setup component includes retention into the donor, membrane absorption, and fluid receptor permeation. The term release is used for the general contextualization of these processes. The results regarding release rate (%/h) and cumulated content of α‐tocopherol (%) are reported.

The 24‐h tests reveal that α‐tocopherol incorporated in microcapsules, with and without Mo extract, exhibited faster release than their non‐coacervated counterparts, with membrane and receptor fluid showing significantly higher α‐tocopherol content for microencapsulated forms (α = 0.05). In the first hour, the highest absorption rate was observed for the MC α‐toc membrane at 0.042%⋅h^−1^, corresponding to the highest permeation of α‐tocopherol into the receptor fluid. However, significant differences between the encapsulated and the free bioactive were observed only after 2 h. Membrane absorptions were 6% for MC α‐toc and 3% for MC Mo + α‐toc, while the nonencapsulated bioactives remained below 1%.

Over time, a decreasing trend in the release rate was observed, though this was not reflected in the cumulated content of α‐tocopherol. A constant permeation was shown in the receptor fluid, whereas the membranes showed enhanced α‐tocopherol absorption. At 24 h, both free bioactives were slightly higher than those in the encapsulated forms.

Overall, the results show that α‐tocopherol is progressively released from the donor chamber, with its content decreasing as membrane absorption and permeation into the receptor fluid increase, particularly for the microencapsulated forms. Moreover, the behavior observed for the microcapsules in the donor chamber suggests a controlled release over time. This is shown by quantifying the contents inside and outside the delivery system. The remaining α‐tocopherol inside the microcapsules was 70% and 82% for MC α‐toc and MC Mo + α‐toc, respectively, while the outside contents fluctuated between 5% and 7%, indicating gradual release of α‐tocopherol.

The cutaneous bioavailability of α‐tocopherol in various cosmetic vehicles, including water‐in‐oil (w/o), oil‐in‐water (o/w) emulsions, as well as liposomes and alcohol‐containing hydrogel formulations, was evaluated using reconstituted human skin models similar to those used in this study [36]. After 24 h of release test, most α‐tocopherol content was absorbed in the epidermis (membrane), mainly from w/o emulsion. However, permeation into the dermis (receptor fluid—PBS buffer solution) was considered irrelevant.

This work has shown significant improvements in the release of α‐tocopherol when using a microencapsulated system to deliver α‐tocopherol through the emulated human skin. These results are comparable to and superior to those observed with its free form, where permeation into the receptor fluid was only detected after 22 h [17].

The validation of the release tests is shown in Table 3. All samples were analyzed throughout the release period and presented in mass and percentage format. The mass balance includes the sum of the α‐tocopherol content quantified in the three components, including outside and inside the microcapsules when applicable. The experimental error generally falls within the accepted range (85% and 115%) defined by the SCCS guidelines [35]. However, some deviations were observed in the MC α‐toc mass balance, with values ranging from 78% to 126%.

**TABLE 3:** Validation of α‐tocopherol release and permeation studies through mass balance based on the Scientific Committee on Consumer Safety (SCCS) guidelines [35].

	*t* (h)	Donor MC (A, A*)					Membrane (B)				Receptor fluid (C)				Mass balance (A = A* + B + C)			
		Mo + α‐toc		α‐toc			Mo + α‐toc		α‐toc		Mo + α‐toc		α‐toc		Mo + α‐toc		α‐toc	
		mg	%	mg	%		mg	%	mg	%	mg	%	mg	%	mg	%	mg	%
Free‐bioactives	0	81.16	100a	81.31	100a	—	0.00	0c	0.00	0d	0.00	0.0c	0.00	0.0d	Sum	Error	Sum	Error
	1	79.19	98a	77.47	95ab		0.66	1bc	1.08	1cd	0.05	0.1b	0.00	0.0d	79.90	102	78.55	103
	2	73.98	91a	74.55	92abc		0.87	1bc	1.17	1cd	0.07	0.1a	0.06	0.1bc	74.92	108	75.79	107
	4	69.61	86a	69.38	85bc		1.19	1bc	2.85	4b	0.06	0.1b	0.08	0.1a	70.85	113	72.30	111
	8	65.78	81a	65.26	80cd		2.97	4b	3.68	5b	0.07	0.1a	0.07	0.1ab	68.82	115	69.01	115
	24	59.22	73a	57.05	70d		9.50	12a	9.34	11a	0.05	0.1b	0.06	0.1c	68.77	115	66.45	103

	*t* (h)	Donor outside MC (A, A*)				Donor inside MC (A, A*)				Membrane (B)				Receptor fluid (C)				Mass balance (A = A* + B + C)			
		Mo + α‐toc		α‐toc		Mo + α‐toc		α‐toc		Mo + α‐toc		α‐toc		Mo + α‐toc		α‐toc		Mo + α‐toc		α‐toc	
		mg	%	mg	%	mg	%	mg	%	mg	%	mg	%	mg	%	mg	%	mg	%	mg	%
Microcapsules	0	0.00	0b	0.00	0b	1.41	100a	1.34	100a	0.00	0a	0.00	0a	0.00	0.0d	0.00	0.0a	Sum	Error	Sum	Error
	1	0.07	5a	0.06	5a	1.39	99a	1.29	96a	0.02	1a	0.06	4a	0.06	3.9bc	0.06	4.2a	1.54	91	1.46	91
	2	0.06	5a	0.10	7a	1.32	93a	1.32	98a	0.04	3a	0.09	6a	0.05	3.7c	0.07	5.0a	1.48	95	1.63	78
	4	0.07	5a	0.07	5a	1.26	89a	1.32	99a	0.05	4a	0.10	8a	0.05	3.7c	0.06	4.4a	1.43	99	1.62	79
	8	0.07	5a	0.07	5ab	1.22	86a	1.32	99a	0.10	7a	0.09	7a	0.06	4.1ab	0.05	4.0a	1.44	98	1.54	85
	24	0.07	5a	0.07	5ab	1.16	82a	0.94	70a	0.10	7a	0.11	8a	0.06	4.2a	0.05	4.0a	1.40	101	0.99	126

*Note:* A: total initial mass; A*: released mass into the donor chamber over time, B: absorbed mass by the membrane over time; and C: mass penetrated the receptor fluid over time. Initial mass: Mo + α‐toc = 81.16 mg; α‐toc = 81.31 mg; MC Mo + α‐toc = 1.41 mg; MC α‐toc = 1.34 mg. Different letters in the same column represent significant statistical differences (α = 0.05).

## 5 Cosmetic Formulations

Two hydrating cream formulations were developed and compared to a commercial product. The new formulations were boosted with MC α‐toc and MC Mo + α‐toc to replace the free form α‐tocopherol in the original formulation. Therefore, this ingredient was occulted from the hydrating creams enriched with microcapsules. This setup was designed to assess the potential of the previously developed delivery systems and the additional effects of Mo, as evaluated through the analytical skin tests described in section 6.

### 5.1 Materials

Cetanol was purchased from Vevy Europe (Genova, Italy), C12 Acid PEG‐8 ester and sodium hyaluronate RM‐CHEM‐10 were acquired from Shijiazhuang (Hebei, China), and citric acid was obtained from Caleo (Graz, Austria). The carbomer Carbopol Ultrez 10 was purchased from Lubrizol (Ohio, United States), 2‐phenoxyethanol, ethylhexylglycerin, and potassium sorbate were obtained from IMD Sweden AB (Malmo, Sweden), butylene glycol was acquired from Gulf Chemical (Mandai, Singapore), and potassium hydroxide from Sigma Aldrich (Missouri, United States). The epidermal growth factor (EGF) was obtained from ORF Genetics (Reykjavík, Iceland).

### 5.2 Hydrating Creams Production

An oil‐free, fast‐absorbing formula was developed and modeled after the commercial version of BIOEFFECT's hydrating cream (control, CF). This product is an emulsion free of fragrance, alcohol, gluten, and parabens, consisting of just sixteen ingredients, including soft Icelandic water, hyaluronic acid, α‐tocopherol, and EGF. To guarantee high‐quality cosmetic products, BIOEFFECT follows the Quality Management System (ISO 9001:2015) according to ISO 22716:2007 Cosmetics GMP guidelines [37] and EU Regulation (EC) No 1223/2009 [9].

**FIGURE 4:**
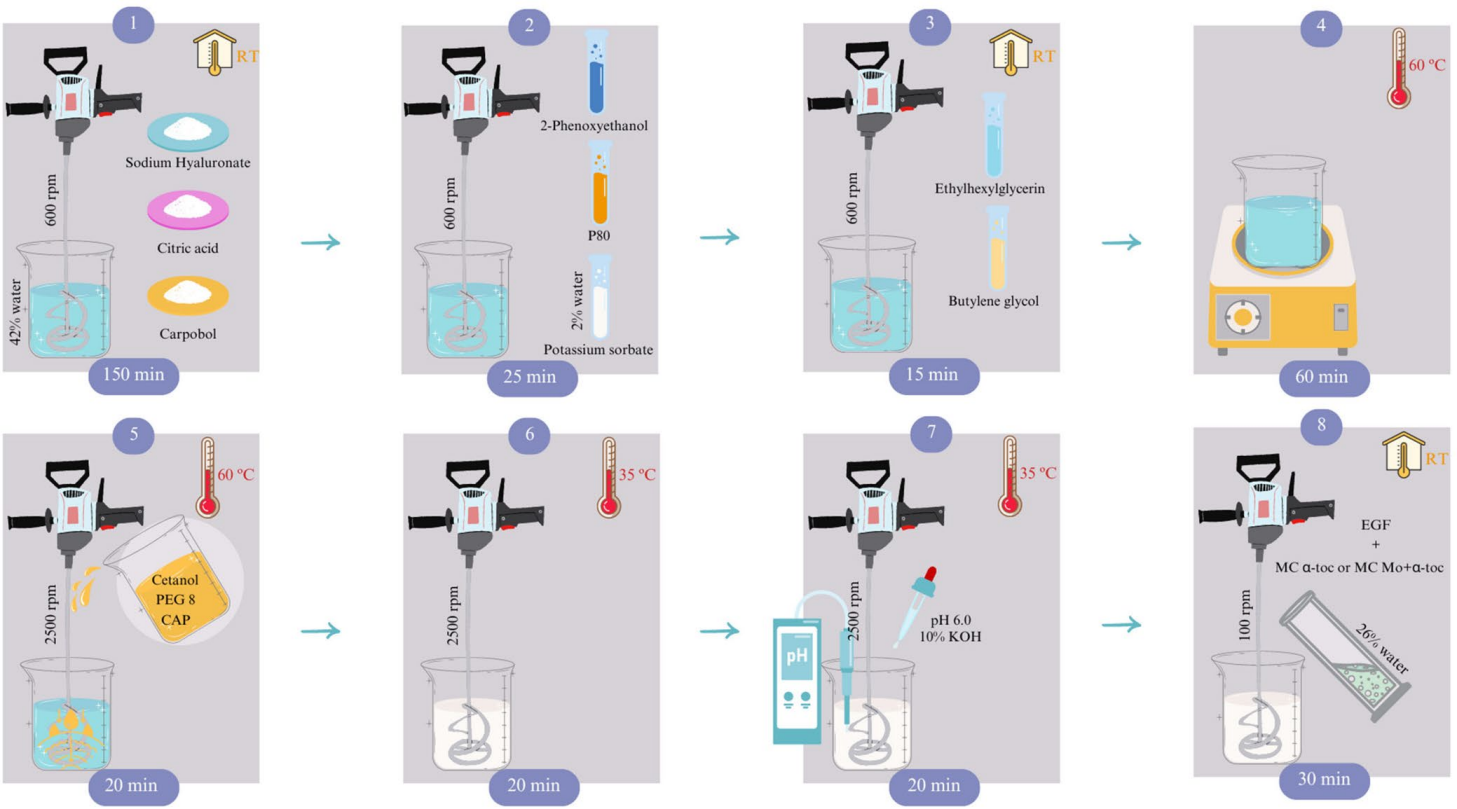
Schematic setup for the development of the cosmetic formulations. Notation in order of appearance: CAP, caprylic triglyceride; EGF, epidermal growth factor; MC Mo + α‐toc, microcapsules of *Moringa oleifera* L. leaf extract and α‐tocopherol; MC α‐toc, microcapsules of α‐tocopherol alone; P80, polysorbate 80; PEG 8, polyoxyethylene glycol; RT, room temperature.

For this study, two versions of the hydrating cream were produced where the free form of α‐tocopherol was omitted, and instead, MC α‐toc or MC Mo + α‐toc were added in the last blending step, as shown in Figure 4.

The procedure is summarized as follows. In the first four steps, the preparation of the water phase consists of (1) mixing the humectant (sodium hyaluronate), pH regulator (citric acid), and thickener (carbopol) in 42% of the total UV filtered water content assisted by a stirrer (Hei‐torque Core, Heidolph, Schwabach, Germany) coupled to a TR 21 radial‐flow impeller (50 mm) at 600 rpm, guaranteeing pH between 3.3 and 3.7 at 20°C using a portable pH meter (Testo 206‐pH 2, Campinas, Brazil); (2) adding a first preservative (2‐phenoxyethanol) and the emulsifier (P80), followed by a second preservative (potassium sorbate) dissolved in 2% of the total water content; (3) adding a third preservative (ethylhexylglycerin) and texture enhancer (butylene glycol); and (4) heating the solution to 60°C. Next, the oil phase preparation is performed separately by mixing the emollients (Cetanol, PEG 8, and CAP) at 60°C. In steps (5) and (6), the oil phase is slowly poured into the water phase and blended at 2500 rpm using a lab shear mixer (AE500S‐H 70G, Huanyu, Wenzhou, China) until the mixture reaches 35°C (circa 40 min total). In step (7), the pH is adjusted to 6.0 using a second chelating agent (KOH), and then the mixture is homogenized over 20 min. Finally, in step (8), EGF and the microcapsule dispersions are added and mixed for 30 min at 100 rpm using a stirrer (Hei‐torque Core, Heidolph, Schwabach, Germany) coupled to a PR 30 pitched‐blade impeller.

These bioactives complete the water phase content, bringing it to a total of 70%. The microencapsulated dispersions introduced into the formulations are estimated based on EE% (section 3.3), ensuring the amount is proportional to its free form in the control formulation (CF) and equivalent to 1%. Additional bioactives present in the Mo extract were omitted for calculation purposes.

Skin compatibility and stability of the hydrating cream formulations were then assessed.

### 5.3 Skin Compatibility Test

An assessment of the skin compatibility of the hydrating creams containing 26% MC Mo + α‐toc in solution was conducted by the certified company SGS Idea (ideatestsgroup.com, Marillac, France). The test involved a simple application under occlusion conditions using a Finn Chamber or equivalent for 48 h under dermatological control. A 20 μL (or 0.02 g) sample was applied on a patch to the external face of the arm at 20°C to evaluate cutaneous compatibility.

Twelve volunteers were recruited for this study, with inclusion criteria specifying healthy participants aged 18–65 years with neither dry skin nor sensitive skin on the tested area. All participants had normal clinical examinations prior to the test, without a strong allergy history and non‐evolutive atopy or dermatological lesions on the tested area, and with no significant history of allergy or irritability to household products. The noninclusion criteria included pregnant or breastfeeding women, volunteers with a general disease incompatibility with the study or active dermatological disease, and volunteers taking anti‐inflammatory drugs, corticosteroids, histamine antagonists, or any other treatment reducing or inhibiting inflammatory or allergic reactions. All volunteers involved in the study agreed to the test conditions.

The test was conducted over a 48‐h period, and the evaluation took place 30 to 40 min after the patches were removed. Clinical assessments were performed to rate cutaneous reactions for erythema and edema. The scale ranged from 0 to 3, where 0 represents no reaction, and 3 is significant alterations. Bubbles, papules, vesicles, dryness, desquamation, and roughness were also considered in the assessment. The mean irritation index (M.I.I.) was calculated by.

(5) $$M.I.I.=\frac{\mathrm{sum}\;\text{of skin reactions}}{\text{number of analyzed volunteers}}$$

Values below or equal to 0.2 are considered nonirritant, while M.I.I. above 2 are classified as very irritant.

According to the results, no formulation has shown irritability after the 48‐h patch test in the twelve healthy volunteers, achieving M.I.I. values ≤ 0.2 (Table 4).

**TABLE 4:** Stability of the hydrating cream formulations containing MC α‐toc and MC Mo + α‐toc, and viscosity and pH of CF.

Test	Compatibility (M.I.I)	Spin	Temperature cycle	Long‐term and heat stability	Viscosity (Pa⋅s)	pH (20°C)
MC α‐toc	Nonirritant (0)	Stable	Stable with slightly different texture	Stable at 4°C and 20°C; unstable at 40°C	1.4ab	5.8–5.9a
MC Mo + α‐toc	Nonirritant (0)	Stable	Stable with slightly different texture	Stable at 4°C and 20°C; unstable at 40°C	1.6a	5.8–5.9a
CF	—	—	—	—	1.2b	5.9–6.1a

*Note:* Criteria: Stable if no phase separation is observed after the stability test; unstable if phase separation is observed after the stability test. Different letters in the same column represent significant differences (α = 0.05).

### 5.4 Stability Tests

Hydrating cream formulations containing MC α‐toc and MC Mo + α‐toc, respectively, were subjected to in‐house stability tests, following the ISO/TR 18811:2018 guidelines for cosmetic product stability [38]. The following tests were conducted:

#### 5.4.1 Spin Test

In each test round, a 40 g sample was placed into a 50 mL falcon tube and incubated in a mini oven (MK II, MWG‐Biotech, Bavaria, Germany) at 37°C for 1 h. After incubation, the sample underwent centrifugation at 300 rpm for 15 min using a benchtop centrifuge (Avanti J‐15R, Beckman Coulter, Indianapolis, United States). This process was repeated for six rounds, with visual inspections after each cycle. The sample was considered stable if no phase separation was observed after all rounds.

#### 5.4.2 Temperature Cycle

A 25 g sample was placed into the final container and exposed to a 4‐day temperature cycle test. Each 24‐h cycle involved incubating the sample at −18°C in a refrigerator (ERB 3030, Electrolux, Stockholm, Sweden), followed by transfer to room temperature (20°C), heating to 37°C in a mini oven (MK II, MWG‐Biotech, Bavaria, Germany), and returning to 20°C for the final 24‐h period before visual inspection and image documentation. The analysis was performed in duplicate, and the sample was considered stable if no phase separation or changes in appearance were observed.

#### 5.4.3 Long‐Term and Heat Stability

Three 25 g samples were placed into different containers and incubated at 4°C in a refrigerator (ERB 3030, Electrolux, Stockholm, Sweden), 20°C or room temperature, and 40°C in an incubator (Hood TH 30, Edmund Buhler, Bodelshausen, Germany). Monthly inspections were performed over 60 days, coinciding with the skin studies described in section 6. These inspections included homogeneity, visual appearance, and odor, all of which were documented with images and records.

#### 5.4.4 pH

A 40 g sample was placed into a 50 mL falcon tube and incubated at 20°C for 60 days. Biweekly, the pH was measured using a portable pH meter (Testo 206‐pH 2, Campinas, Brazil) and recorded. The test was performed in triplicate for each formulation and CF. The products were considered stable if the pH remained within 6.0 ± 0.3.

#### 5.4.5 Rheology

Viscosity and thixotropy effects were monitored using a coaxial rheometer (RST‐CC, Brookfield, Toronto, Canada). A 0.8 mL sample of the CF and MC formulations was subjected to shear rates ranging from 30 to 360 s^−1^, and shear stresses from 240 to 720 Pa. The equipment operated at 20°C with a parallel plate geometry and a gap of 0.5 mm, using Rheo3000 software for PC control and data acquisition. ANOVA and Tukey tests were used to assess the significant differences with α = 0.05 (see section 3.3).

Table 4 shows stability test results for the hydrating cream formulations containing MC α‐toc and MC Mo + α‐toc, with visual data provided in Figure S1. The formulations were stable for the spin test and temperature cycle. However, slight color differences were observed after exposure to high temperatures (Figure S1a,ii and b,ii). Initially, the hydrating creams presented a white‐like color and soft texture and were fragrance‐free. These characteristics were preserved during the long‐term and heat stability for 4°C and 20°C, conditions accepted for the storage of emulsions. At 40°C, the formulations showed destabilization of the oil–water interface due to the elevated temperatures. This is an expected result as temperature, rheological profile, and pH play a crucial role in cosmetic stabilization [39]. Both viscosity and pH were also evaluated and compared to CF. MC Mo + α‐toc exhibited a significant difference in viscosity, although no statistical difference in pH was observed, with values remaining within the desired range of 5.7–6.3. This pH ensures non‐irritancy and biocompatibility toward the application [40] and belongs to the pH range of creams produced with Mo leaf extracts (5.6–7.0) [41]. Visual inspections revealed no notable differences in texture or odor across all samples, consistent with CF and literature [41].

## 6 Cosmetic Creams Performance Test

### 6.1 Volunteers Selection

Thirty healthy Icelandic participants, female and male, aged 20 and older, enrolled in the skin performance study between March and May 2024. They were equally distributed into three groups, with 10 volunteers for each formulation: MC α‐toc, MC Mo + α‐toc, and CF. The study was conducted over 60 days and included three visits to the Skin Lab at BIOEFFECT HQ. Volunteers were selected, recruited, and screened for eligibility using a standard in‐house selection procedure.

Exclusion criteria included participants with known sensitivities to any ingredients in the formulation or those with skin diseases affecting the facial area. To ensure consistency, volunteers planning to travel to warmer climates during the study period were excluded, as well as those who had undergone facial cosmetic procedures (such as surgical procedures, laser treatments, injections with dermal fillers or toxins, microneedling, or similar) in the 12 months prior to enrolment or planned to undergo these treatments during the study. Additionally, participants who had used BIOEFFECT skincare products containing EGF in the 12 months prior to enrolment were also excluded. Following consultation, the Icelandic Ethical Committee confirmed that BIOEFFECT's skin assessment studies fall outside the scope requiring formal ethical review. This is due to the absence of biological sampling, the use of nonpharmaceutical, nonmedical products, and the fact that the studies are not intended for diagnosing or treating any health conditions. All participants gave written consent allowing the use of their images for research‐related purposes.

### 6.2 Analytical Skin Assessment

Following selection, the participants visited the Skin Lab to provide informed consent and participate in the baseline measurement. They were instructed to apply the product twice daily, in the morning and evening, while avoiding the use of any other regular skincare products such as hydrating creams, serums, and eye creams. Sunscreen use was recommended during sun exposure. The volunteers attended two additional visits for skin evaluations at 30‐day intervals.

Participant satisfaction and tolerability were recorded throughout the study. At each visit, the researcher assessed and documented the effects on the participants' skin using the following two pieces of equipment designed to analyze various skin parameters.

#### 6.2.1 Canfield VISIA‐CR

The Canfield VISIA‐CR Imaging System (Parsippany, United States) was used to quantitatively analyze various skin parameters. This equipment features a photo booth that captures a sequence of digital images using three unique lighting methods: standard white light, cross‐polarized light, and UV fluorescence (Figure S2a).

Volunteers placed their faces in the booth to register and analyze all visible changes before and after product use. The regions analyzed in this test were the forehead and both cheeks, where the following parameters were evaluated: skin texture, pigmentation spots (brown spots and spots), pores, UV damage, red areas (acne, inflammation, rosacea, or spider veins), wrinkles, and fine lines.

**FIGURE 5:**
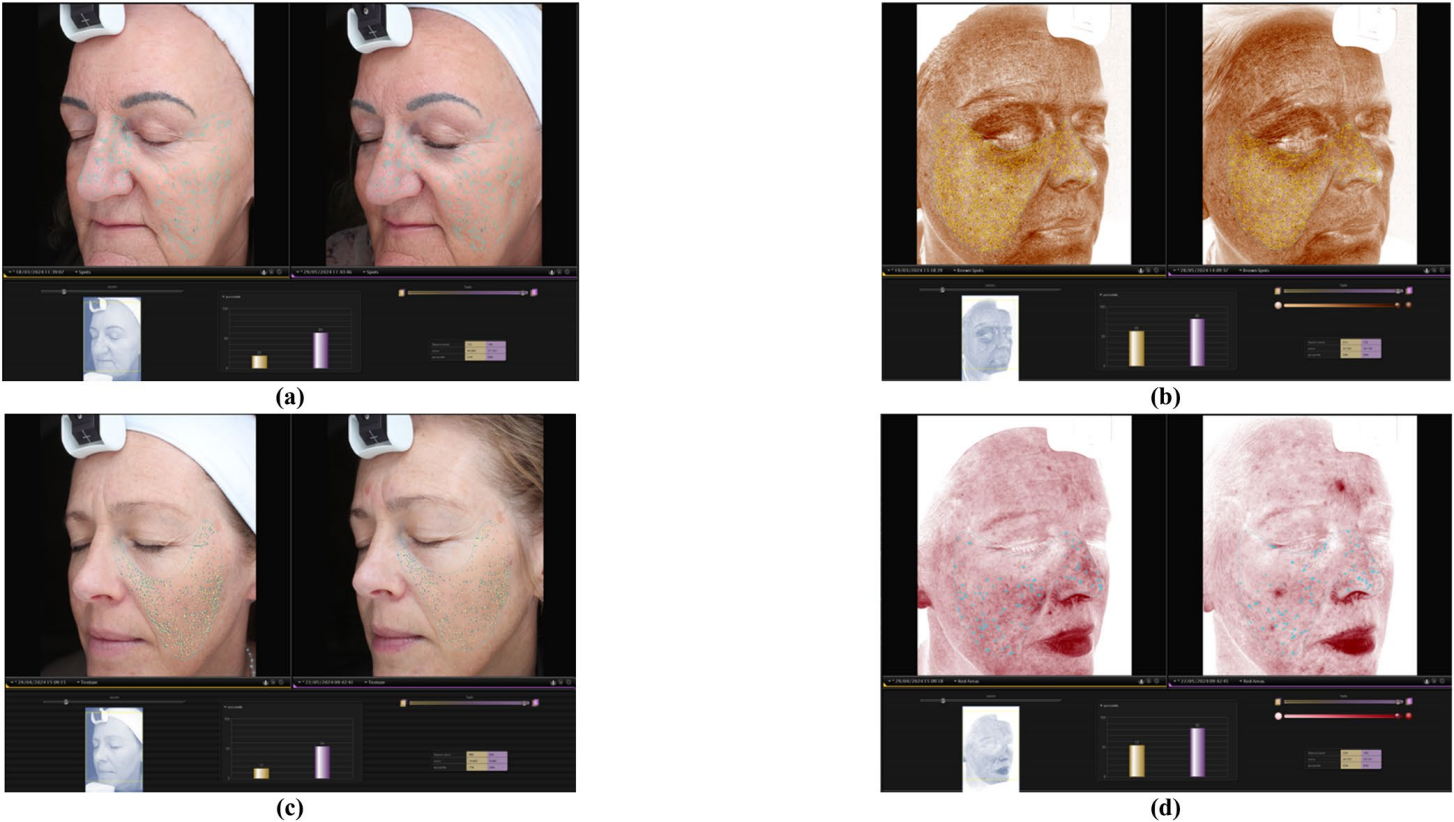
Examples of skin measurements performed over the 30 and 60‐day study period, evaluating (a) spots and (b) brown spots in volunteers using MC α‐toc hydrating cream, and (c) texture and (d) red areas in volunteers using MC Mo + α‐toc product.

Figure 5 illustrates the images captured using the Canfield VISIA‐CR, analyzing four parameters with their respective improvements over time displayed in the bar graph below. The percentages indicate the degree of improvement compared to the volunteer's average skin type and age. The yellow bars represent the initial measurement, while the purple bars correspond to the results after product use.

#### 6.2.2 Cortex DermaLab

The Cortex DermaLab Combo 4 Skin Analysis System (Aalborg, Denmark) was used to measure various skin parameters, including skin hydration, elasticity, density (using ultrasound), and transepidermal water loss (TEWL). Each parameter was assessed using specific analysis probes, as shown in Figure S2b–f. In these tests, only the cheek area was analyzed.

**FIGURE 6:**
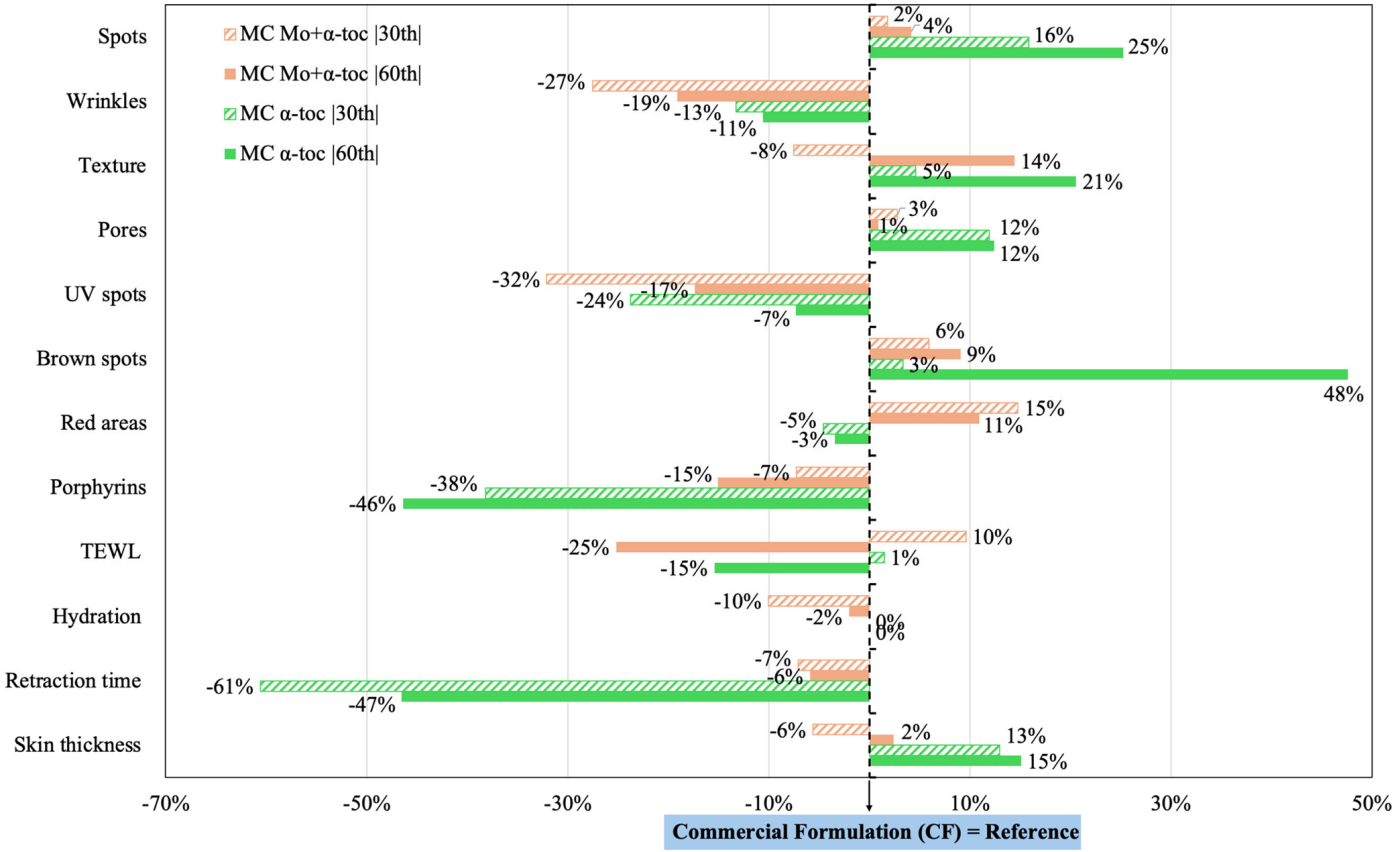
Performance of the hydrating creams containing MC α‐toc and MC Mo + α‐toc, standardized and compared to the commercial formulation. Skin measurements performed at 30 and 60‐day product use with 30 volunteers.

The efficacy of the hydrating creams containing MC α‐toc and MC Mo + α‐toc was tested in human skin and compared to those obtained for the CF. The three formulations were set in a similar environment for evaluation, and the representative data was standarized to avoid skin type and initial skin conditions interference. Figure 6 shows the results of the measurements performed after 30 and 60‐day applications, where CF is indicated as a reference for all parameters evaluated using both Canfield VISIA‐CR and Cortex DermaLab equipments.

Considerable improvements were observed across most parameters, including a 25% reduction in spots with MC α‐toc, texture improvements of 14% with MC Mo + α‐toc and 21% with MC α‐toc, a 12% reduction in pores with MC α‐toc, a 48% reduction in brown spots with MC α‐toc, and an 11% reduction in red areas with MC Mo + α‐toc. Notable effects were also seen in transepidermal water loss (TEWL) and retraction time (skin firmness), with reductions of 25% for MC Mo + α‐toc and 47% for MC α‐toc, respectively. Additionally, MC α‐toc led to a 15% increase in skin thickness compared to CF.

Despite the similar composition of the microcapsules, the delivery systems affected human skin differently. MC α‐toc was more effective in reducing spots and brown spots, highly related to skin pigmentation. At the same time, MC Mo + α‐toc improved skin appearance by decreasing red areas, a reference to acne, inflammation, rosacea, or spider veins. The reduction of red areas may be linked to the anti‐inflammatory properties of Mo leaves, as discussed in prior reports [12, 42, 43]. Other benefits of α‐tocopherol and Mo extract were mentioned in delaying photoaging and reducing the associated oxidative stress [42].

Previous cosmetic products developed using Mo were designed for UV protection due to the high antioxidant activity of this crop material [3]. When 5% of the extract was incorporated into an o/w emulsion, the authors observed about 50% efficacy against UVB radiation. In this work, UV spots were not an improved parameter compared to CF. CF also outperformed in reducing wrinkles and porphyrins. While the reasons for these outcomes are not entirely clear, the lower pH of the newly developed hydrating creams might reduce their effectiveness in minimizing wrinkles. However, this did not affect the measured hydration levels. The poor performance of both MC formulations in reducing porphyrins, which are associated with bacterial excretions that can cause acne, might be due to a higher concentration of polymers on the skin. This can occur because the microcapsules are deposited on the skin to facilitate the controlled release of the active ingredients.

During the skin studies, weather changes were observed from March to May 2024 due to the seasonal transition in Iceland. According to the local forecast, temperatures ranged from −1.6°C to 6.9°C, while air humidity varied from 59% to 78% [44]. These factors were directly related to the extreme weather conditions the volunteers experienced. However, this did not hinder their ability to engage in outdoor activities, such as sea swimming, which were frequently mentioned during the three visits.

### 6.3 Self‐Assessment Study

An electronic self‐assessment questionnaire was administered after 60 days of product use to complete the skin studies. The questionnaire captured participants' perceptions of their facial skin appearance, the perceived effectiveness of the study product, and global satisfaction across 16 questions. Responses were scored on a scale of 1–5, where 1 represented “disagree” and 5 represented “completely agree”. Additionally, the evaluation and reporting of any adverse events were carried out throughout the study period.

**FIGURE 7:**
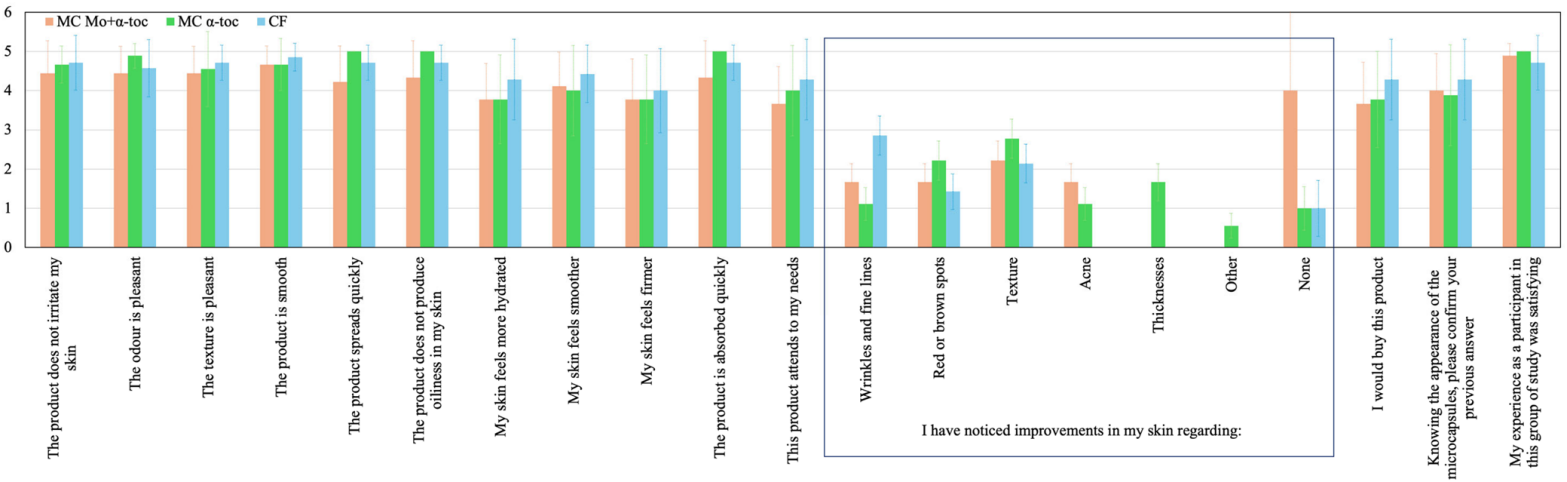
Cosmetic acceptance obtained by the self‐assessment study of the hydrating creams containing MC Mo + α‐toc, MC α‐toc, and CF after 60‐day product use. Scores: 1 = disagree; 5 = completely agree.

Overall, the incorporation of microcapsules significantly enhanced the performance of the hydrating creams. As shown in Figure 7, the self‐assessment results for the three formulations demonstrate a high level of volunteer acceptance. Despite being shown confocal images of the microcapsules (available in [15]), volunteers maintained positive perceptions. The evaluation covered various aspects of the products, including their characteristics and performance on the skin. Participants rated their level of agreement on a scale from 1 to 5, with an average score around 4. No sensory differences were observed between the microcapsule‐containing formulations and the commercial control product. All products were perceived as smooth, with a pleasant fragrance and texture, easy to apply, and rapidly absorbed into the skin without irritating effects. Participants appreciated their involvement in the skin study, reporting that their skin required a brief adaptation period, with more pronounced improvements noted as they continued using the products.

## 7 Conclusions

This study demonstrated the potential of microcapsules containing α‐tocopherol (MC α‐toc) and *Moringa oleifera* Lam. (Mo) leaf extract with α‐tocopherol (MC Mo + α‐toc) to enhance the performance of hydrating creams compared to a commercial formulation (CF).

The supercritical carbon dioxide extraction of Mo leaf extract, performed under optimized conditions, yielded 8.43% ± 0.09% relative composition and 232.5 ± 3.2 mg_α‐tocopherol_⋅g_extract_^−1^, underscoring its potential as a cosmetic ingredient. This extract and α‐tocopherol were microencapsulated to increase stability and enhance the permeation to the skin. The microcapsules MC α‐toc and MC Mo + α‐toc showed high encapsulation efficiencies of 95.1% ± 1.2% and 93.4% ± 1.3%, with loading contents of 10.3% ± 1.2% and 10.0% ± 1.3%, respectively. These results underscore the effectiveness of the coacervation process in incorporating hydrophobic bioactives.

Franz cell studies revealed that MC α‐toc and MC Mo + α‐toc significantly increased the release rates of α‐tocopherol through simulated human skin compared to their nonencapsulated counterparts. MC α‐toc demonstrated the highest absorption rate into the membrane with 0.042%·h^−1^ during the first hour, also indicating enhanced permeation of α‐tocopherol into the receptor fluid. After 2 h, cumulative membrane absorption was 6% for MC α‐toc and 3% for MC Mo + α‐toc, while nonencapsulated α‐tocopherol remained below 1%. Although the release rate decreased over time, the cumulated content of α‐tocopherol remained steady over a 24‐h period, demonstrating a controlled release mechanism.

Additionally, two stable and nonirritant hydrating creams incorporating these microcapsules were formulated and tested against BIOEFFECT's commercial formulation (CF) in a 60‐day study with 30 Icelandic volunteers. The creams containing the microcapsules showed substantial improvements across various skin parameters, including increased skin elasticity and thickness, and improved pigmentation and texture. Specifically, MC α‐toc reduced pigmentation by 25% in spots and 47% in brown spots. In comparison, MC Mo + α‐toc was more effective in reducing red areas associated with skin inflammation and improving skin texture by 11% and 15%, respectively. Despite Iceland's challenging climatic conditions, volunteers reported high satisfaction with the product characteristics, including texture, odor, and ease of application, with no irritation observed.

Overall, these findings suggest that microencapsulation is a promising strategy for enhancing the stability and effectiveness of cosmetic formulations, offering significant potential for further innovation in skincare products. Mo, with its rich bioactive profile, stands out as a valuable ingredient in sustainable skincare solutions, offering not only functional benefits but also a natural approach to enhancing skin health and hydration.

## Conflicts of Interest

The authors declare no conflicts of interest.

## Supporting information


**Figure S1:** jocd70486‐sup‐0001‐FigureS1.docx.

**Figure S2:** jocd70486‐sup‐0002‐FigureS2.docx.

## Data Availability

The data that support the findings of this study are available from the corresponding author upon reasonable request.
