# Correction to “Microencapsulated α‐Tocopherol and Moringa Extract for Improved Skin Protection: Insights From Human Skin Assessment in Cosmetic Formulations”

**DOI:** 10.1111/jocd.70557

**Published:** 2025-11-20

J. C. Kessler, I. M. Martins, Y. A. Manrique, et al., “Microencapsulated α‐Tocopherol and Moringa Extract for Improved Skin Protection: Insights From Human Skin Assessment in Cosmetic Formulations,” *Journal of Cosmetic Dermatology* 24, no. 10 (2025): e70486, https://doi.org/10.1111/jocd.70486.

The publication lacks the Acknowledgments section. The correct text is as follows:

“This work was financially supported by Fundação para a Ciência e a Tecnologia, I.P. /MCTES through national funds: LSRE‐LCM, UID/50020/2025; and ALiCE, LA/P/0045/2020 (DOI: 10.54499/LA/P/0045/2020); CIMO, UIDB/00690/2020 (DOI: 10.54499/UIDB/00690/2020) and UIDP/00690/2020 (DOI: 10.54499/UIDP/00690/2020); and LA SusTEC, LA/P/0007/2021 (DOI: 10.54499/LA/P/0007/2020). Júlia Cristiê Kessler acknowledges her PhD scholarship (ref. 2020.06656.BD, DOI: 10.54499/2020.06656.BD) from FCT.

All experimentation presented in Sections 5 and 6 was conducted at BIOEFFECT ehf. (bioeffect.com, Reykjavík, Iceland), inspired by the in‐house methodologies under an agreement established between BIOEFFECT and the Faculty of Engineering of the University of Porto. The authors extend their gratitude to BIOEFFECT for their invaluable support during J.C. Kessler's stay at their facility.”

We apologize for this error.

