# Supplementary material for: Microencapsulated α‐Tocopherol and Moringa Extract for Improved Skin Protection: Insights From Human Skin Assessment in Cosmetic Formulations
Source: J Cosmet Dermatol. 2025 Oct 13;24(10):e70486. doi: 10.1111/jocd.70486 (PMC12516939; doi:10.1111/jocd.70486)
Supplement: Supplementary file 1 — Figure S1: jocd70486‐sup‐0001‐FigureS1.docx. [file JOCD-24-e70486-s001.docx]

**Figure S1.** Stability of the hydrating cream formulations containing (a) MC Mo+α-toc and (b) MC α-toc, in which (i) spin; (ii) temperature cycle; and (iii) long-term and heat stability tests.

| **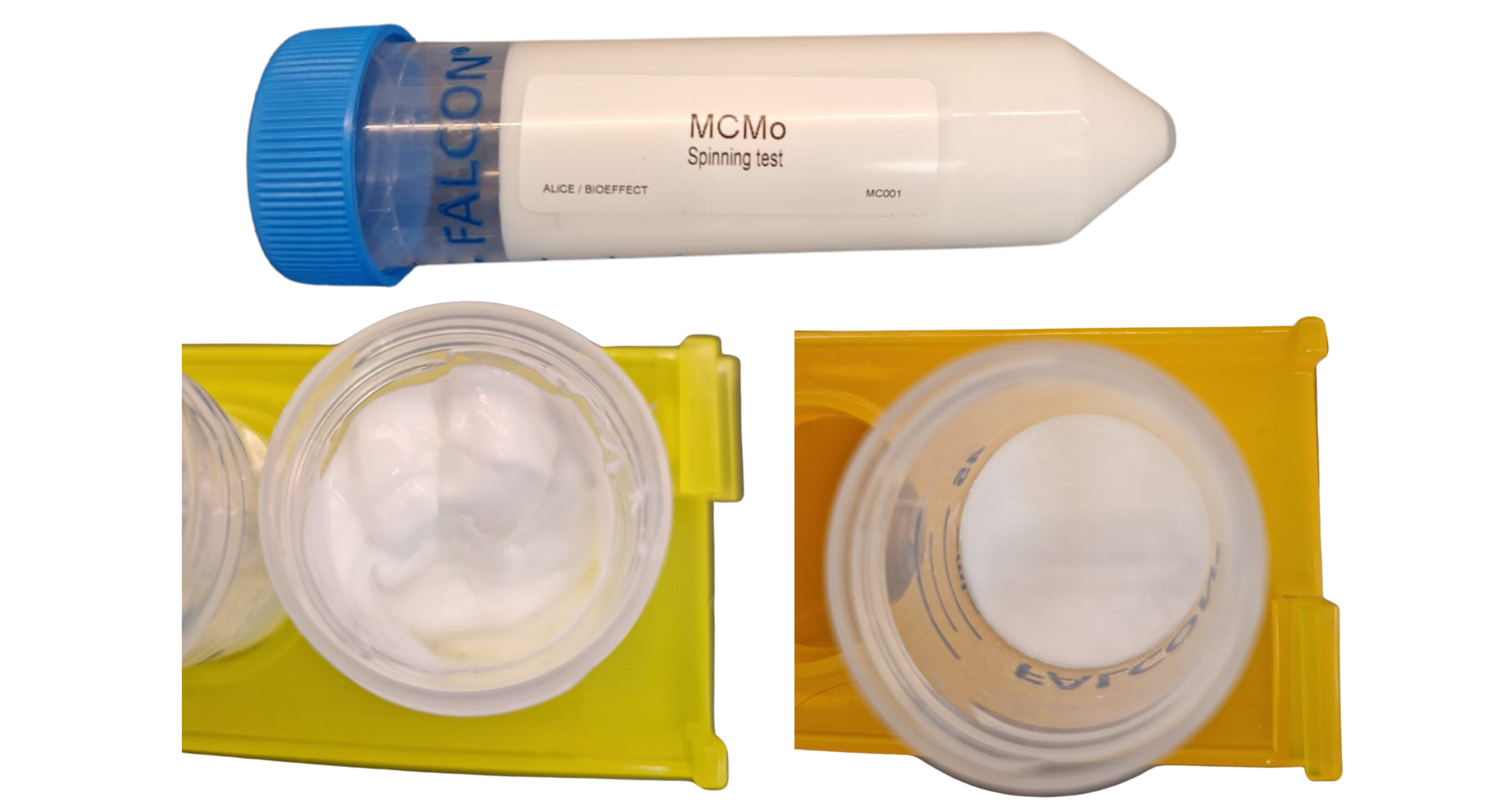**  (a,i)  t_final_  t_initial_ | 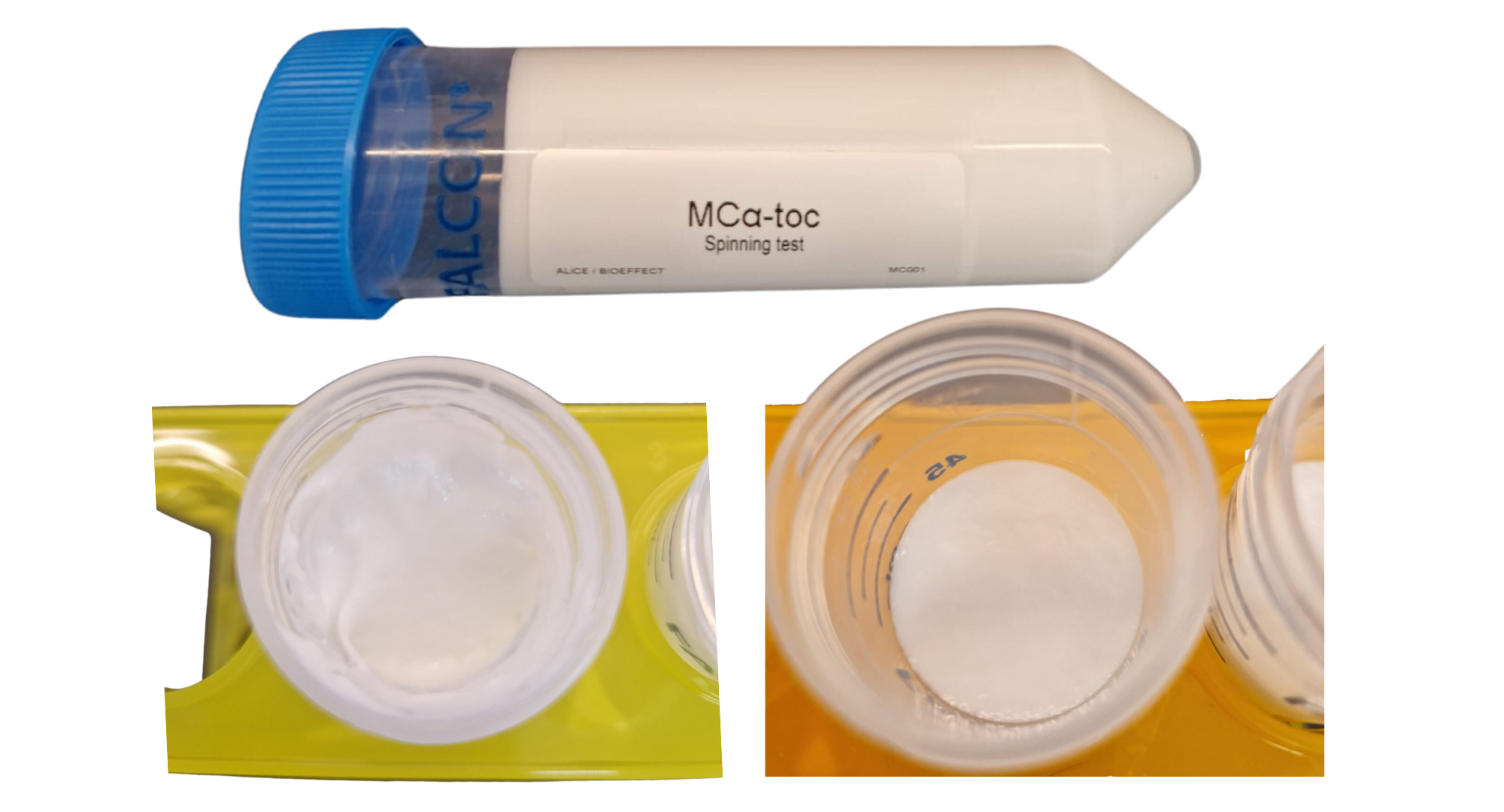  (b,i)  t_final_  t_initial_ |
| --- | --- |
| **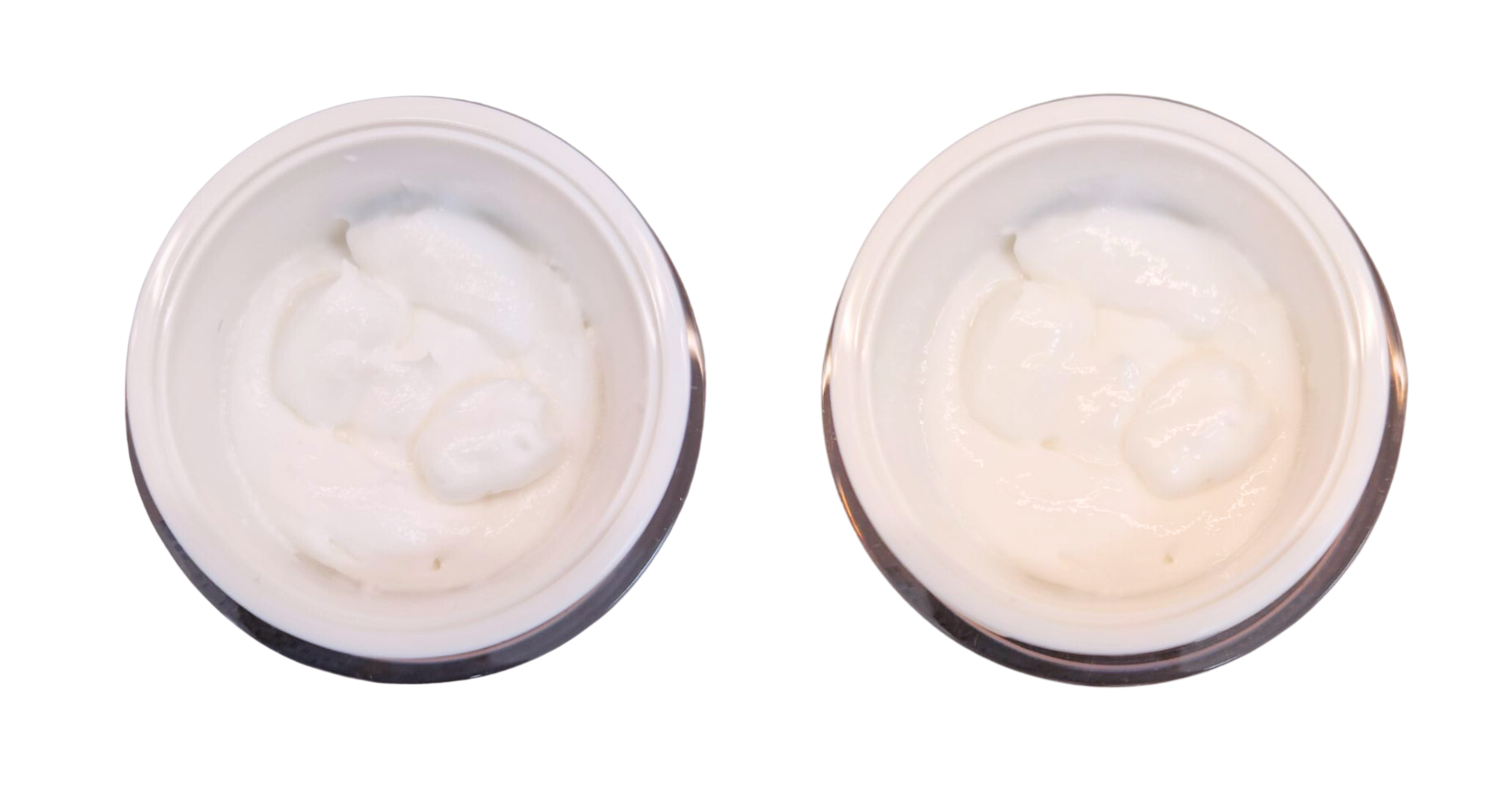**  (a,ii)  t_initial_  t_final_ | **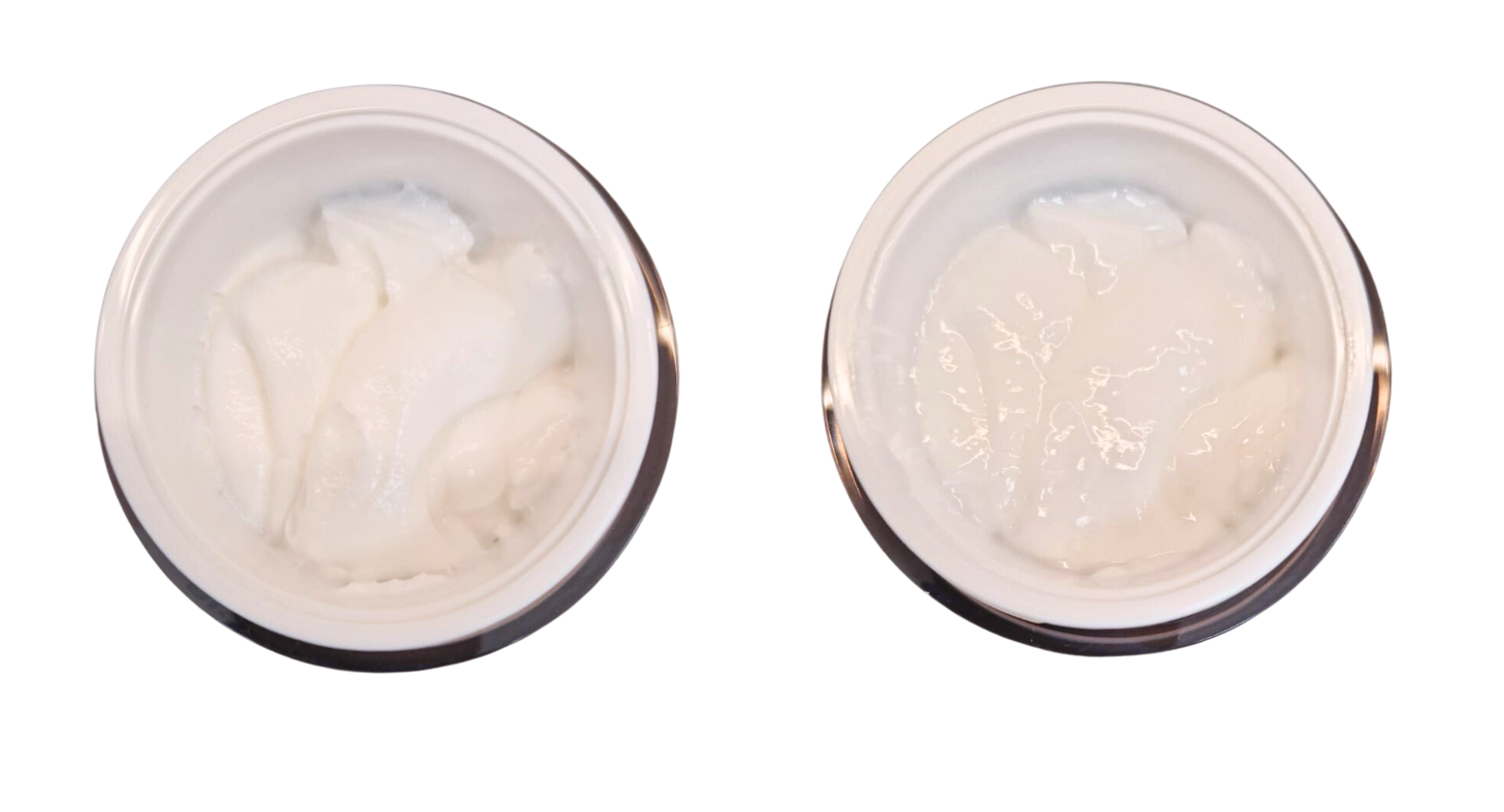**  (b,ii)  t_final_  t_initial_ |
| (a,iii)  20 °C  4 °C  40 °C  t_60day_  t_0day_  / | (b,iii)  4 °C  40 °C  20 °C  t_0day_  t_60day_ |
