# Supplementary material for: Microencapsulated α‐Tocopherol and Moringa Extract for Improved Skin Protection: Insights From Human Skin Assessment in Cosmetic Formulations
Source: J Cosmet Dermatol. 2025 Oct 13;24(10):e70486. doi: 10.1111/jocd.70486 (PMC12516939; doi:10.1111/jocd.70486)
Supplement: Supplementary file 2 — Figure S2: jocd70486‐sup‐0002‐FigureS2.docx. [file JOCD-24-e70486-s002.docx]

**Figure S2.** (a) Canfield VISIA-CR, (b) Cortex DermaLab setup and (c-f) Cortex DermaLab probes used for the skin measurements.

| 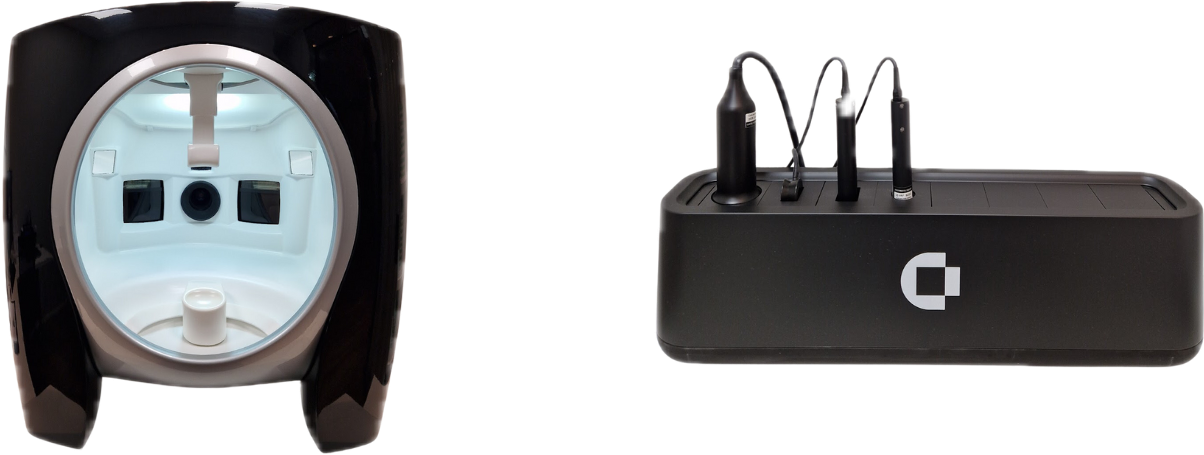 | **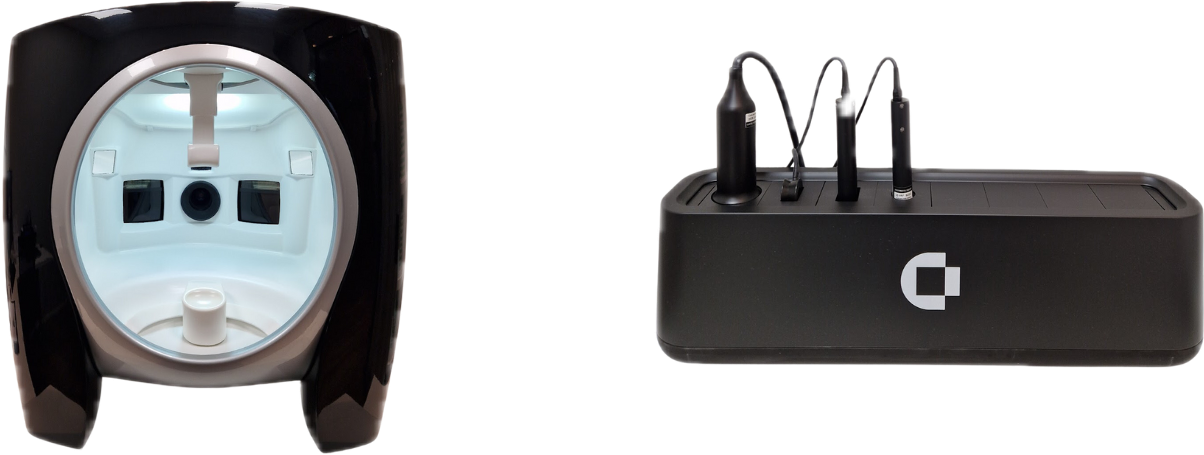** |
| --- | --- |
| **(a)** | **(b)** |
| 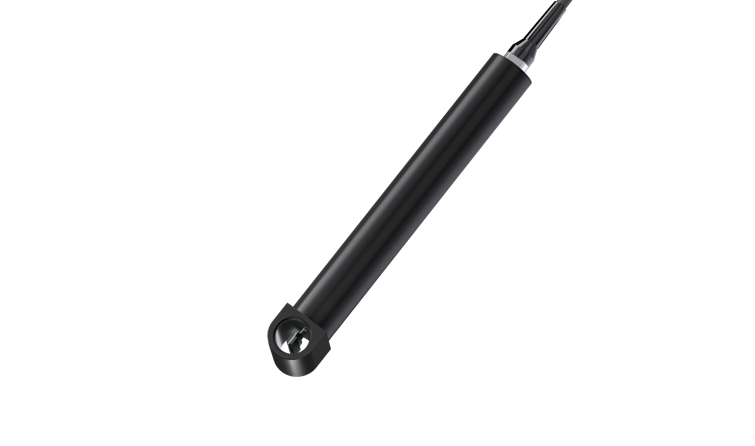  The **TEWL probe** measures the transepidermal water loss, which is the evaporation rate of water through the outer layer of your skin, epidermis. | 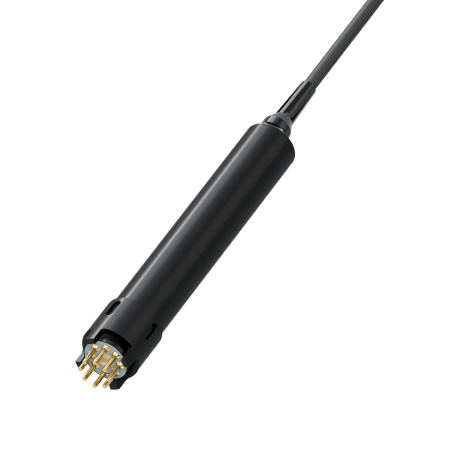  The **Hydration Pin probe** is used to measure hydration - the water binding capacity of the outermost layer of the skin. As a stand-alone measurement or before and after a skin hydration treatment |
| **(c)** | **(d)** |
| 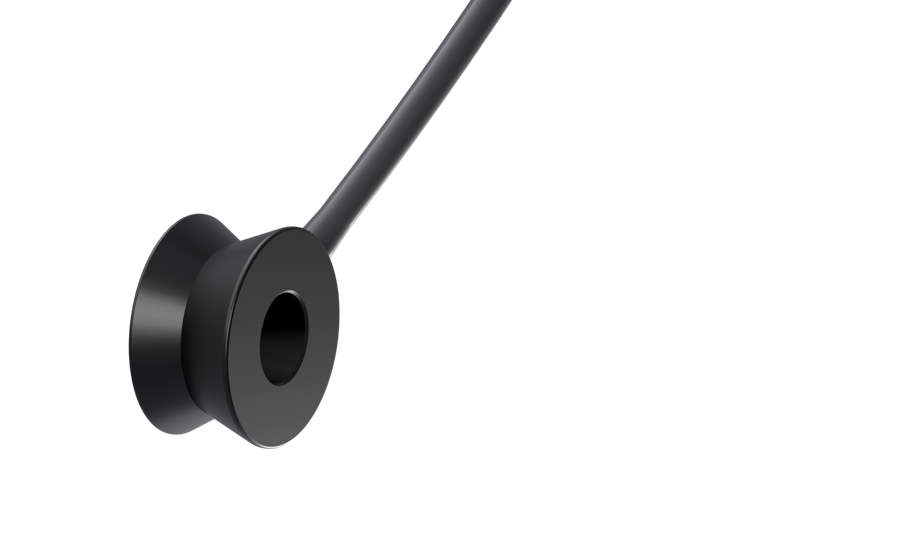  The **Elasticity probe** is based on the suction cup method, which measures the actual elevation of the skin, as a function of a predefined negative pressure setting. | 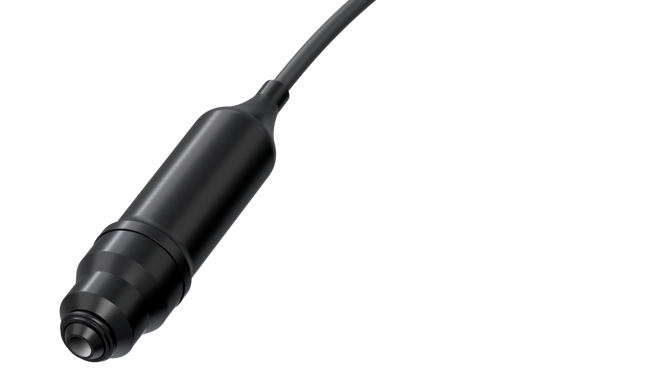  The **Ultrasound probe** provides objective and reliable measurement of the skin’s collagen level and dermal thickness of the skin. |
| **(e)** | **(f)** |
